# Using singscore to predict mutation status in acute myeloid leukemia from transcriptomic signatures

**DOI:** 10.12688/f1000research.19236.3

**Published:** 2019-10-14

**Authors:** Dharmesh D. Bhuva, Momeneh Foroutan, Yi Xie, Ruqian Lyu, Joseph Cursons, Melissa J. Davis

**Affiliations:** 1 Bioinformatics Division, Walter and Eliza Hall Institute of Medical Research, Parkville, VIC, 3052, Australia; 2 School of Mathematics and Statistics, University of Melbourne, Parkville, VIC, 3010, Australia; 3 Department of Clinical Pathology, The University of Melbourne Centre for Cancer Research, Victorian Comprehensive Cancer Centre, Parkville, VIC, 3000, Australia; 4 Department of Medical Biology, University of Melbourne, Parkville, VIC, 3010, Australia; 5 Department of Biochemistry and Molecular Biology, Faculty of Medicine, Dentistry and Health Sciences, University of Melbourne, Parkville, VIC, 3010, Australia

**Keywords:** single sample, gene set scoring, signature scoring, AML mutations, NPM1c mutation, mutation prediction, TCGA

## Abstract

Advances in RNA sequencing (RNA-seq) technologies that measure the transcriptome of biological samples have revolutionised our ability to understand transcriptional regulatory programs that underpin diseases such as cancer. We recently published singscore - a single sample, rank-based gene set scoring method which quantifies how concordant the transcriptional profile of individual samples are relative to specific gene sets of interest. Here we demonstrate the application of singscore to investigate transcriptional profiles associated with specific mutations or genetic lesions in acute myeloid leukemia. Using matched genomic and transcriptomic data available through the TCGA we show that scoring of appropriate signatures can distinguish samples with corresponding mutations, reflecting the ability of these mutations to drive aberrant transcriptional programs involved in leukemogenesis. We believe the singscore method is particularly useful for studying heterogeneity within a specific subsets of cancers, and as demonstrated, we show the ability of singscore to identify where alternative mutations appear to drive similar transcriptional programs.

## Introduction

The development of microarrays and more recently the rapid uptake of RNA-sequencing technologies have provided a platform to examine the transcriptional profile (or transcriptome) of biological samples ^1^. Transcriptomic analyses have traditionally focused on ‘differential expression’ of genes between sets of samples, however with the rapid growth of publicly available RNA data there has been increasing usage of ‘relative approaches’, which quantify the relative concordance of a sample or samples with a specific gene signature ^1^. While sequencing of genomic mutations has been important for classifying different tumour subsets based upon the presence of mutations or fusion genes, and identifying genetic lesions which may act as drivers of cancer progression, transcriptomic profiling can provide further information on the state or phenotype of cells carrying these mutations. Cancers are a heterogeneous set of diseases with a number of clinical and pathological subtypes. In diseases such as breast cancer the primary clinical classifications relate to the expression of hormone receptors (estrogen receptor: ER; and progesterone receptor: PR) or the overexpression of Erb-B2 receptor tyrosine kinase ( *HER2*), as these features can be directly targeted with therapeutic agents. A common example of transcriptomic or gene expression data informing clinical practice is the use of prediction analysis of microarray 50 (PAM50) signatures for distinguishing the intrinsic breast cancer subtypes [ 2, Cieślik and Chinnaiyan ^1^]. For many other cancers, subtype classification has largely relied upon identifying sets of recurrent mutations across large patient cohorts, with whole genome or whole exome sequencing studies helping to resolve the clinically significant subtypes [ 3, Papaemmanuil *et al*. ^4^].

Perhaps the most well-known ‘relative approach’ is single-sample gene set enrichment analysis (ssGSEA) ^5^, often used through the GenePattern web-tool. Another common approach is gene set variation analysis (GSVA) ^6^ which is available as an R/Bioconductor package that also includes functionality for ssGSEA, an alternative approach known as PLAGE ^7^, and a z-score based approach ^8^. Both ssGSEA and GSVA use a Kolmogorov-Smirnov like random-walk statistic to convert normalised gene ranks to the resulting score, however this normalisation procedure means that the scores are not truly ‘single-sample’, and variations in the overall sample composition for a study (e.g. variations in the presence or relative frequency of different cancer subtypes) can lead to unexpected changes in sample scores. Furthermore, the resultant scores from these methods can vary in their range and absolute value, making them difficult to interpret without further processing. To overcome this, we have developed a single-sample gene set scoring method singscore ^9^ which simply uses the ranks of genes within a given set, normalised relative to the maximum and minimum theoretical scores for a gene set of a given size.

Through large scale efforts such as The Cancer Genome Atlas (TCGA), transcriptomic data are available for thousands of clinical samples, often together with corresponding genomic or epigenomic (often DNA methylation) data. These transcriptomic data can help to characterise the functional effects of corresponding mutations, and provide a window to study the heterogeneity which arises within different subtypes of cancer due to epigenetic and transcriptional regulatory programs which can also influence cell behaviour. Here, we demonstrate that the single-sample gene set scoring method singscore ^9^ can be used to classify TCGA AML samples using transcriptional ‘gene signatures’ for the NPM1c mutation, *KMT2A* ( *MLL*) gene fusions, and *PML-RARA* gene fusions that were derived from independent studies. Without any need for parameter fitting or estimation, we show that gene set scoring with singscore can distinguish samples carrying these mutations. The case studies we present demonstrate the application of gene set scoring to examine not only the differences, but also the relative similarities between established subtypes of AML that impact clinical outcome. This workflow is available as a bioconductor workflow package from https://bioconductor.org/packages/release/workflows/html/SingscoreAMLMutations.html.

## Description of the biological problem

As with most cancers, acute myeloid leukemia (AML) is a heterogeneous disease with a number of classified subtypes. Analysis of TCGA AML genomic data identified a number of subtypes based upon the presence or absence of specific ‘driver mutations’; recapitulating and expanding upon previously identified clinical subsets ^3^. A more recent study which focused primarily on genomic data has further refined the clinically significant AML subtypes ^4^, highlighting a number of co-occurring as well as mutually exclusive mutations. As the identification of putative driver fusions/mutations continues, work has also been directed towards how these lesions interact with each other and other features (e.g. cellular proliferation, changes due to phenotypic plasticity, or variation in post-transcriptional regulators such as microRNAs) to drive transcriptional changes as discussed in a recent review ^10^.

Of note for this work, one of the most common mutations in clinical AML samples is a frameshift mutation within exon 12 of the nucleophosmin ( *NPM1*) gene ^4^. This mutation leads to aberrant localisation of nucleophosmin with cytoplasmic accumulation rather than localising to the nucleolus, and accordingly this mutation is often referred to as the NPM1c mutation ^11^. As noted by Verhaak *et al*. ^12^, the NPM1c mutation is associated with dysregulated activity of the homeobox domain (Hox) family of transcription factors which are essential for developmental patterning. The effects of this mutation in disease progression have been further demonstrated in recent work which showed that loss of NPM1c leads to differentiation of AML cells ^11^.

Further recurrent genetic lesions in AML relevant for this work include lysine methyl transferase 2A ( *KMT2A*; previously known as *MLL*) fusion genes, partial tandem duplications within *KMT2A* ( *KMT2A*-PTD), and fusion genes between promyelocytic leukemia and retinoic acid receptor alpha ( *PML-RARA*). Given the role of NPM1c in dysregulating the Hox gene family, it is interesting to note that AML samples with MLL fusion genes also show dysregulated expression of Hox family genes [ 13, Ross et al. ^14^]; however, samples with *MLL*-PTD appear to show a relatively distinct phenotype from MLL-fusion samples ^14^. While there is good evidence demonstrating the role of NPM1c mutations and other genetic lesions in blocking AML cell differentiation, the *PML-RARA* fusion subset is diagnostic for a specific subset of AML known as acute promyelocytic leukemia (APL). This clinically distinct subtype of AML is associated with a specific morphology under the French-American-British (FAB) classification of AML, FAB-M3, with cells showing a distinct morphology due to a differentiation block at the promyelocyte stage ^15^.

In this workflow we demonstrate the ability of the singscore method for single sample gene set scoring ^9^ to classify tumour ‘driver mutations’ from transcriptomic data. We use a previously identified gene signature for the NPM1c mutation ^12^. We also use signatures for *PML-RARA* gene fusions and MLL-fusions that were derived using pediatric AML samples but shown to work well for classifying adult AML samples with similar lesions ^14^, although we note that there is evidence of relatively large differences in the mutational profiles of adult and pediatric cancers ^16^. Using these signatures, which are included within the molecular signatures database (MSigDB) ^17^, we demonstrate that a bi-directional scoring approach can classify TCGA AML samples with corresponding mutations with a good precision and recall. A particularly useful feature of gene set scoring is the ability to project samples onto 2D or higher-order landscapes defined by corresponding phenotypic signatures. Accordingly, by comparing scores for both the NPM1c and *KMT2A*-/ *MLL*-fusion signatures, we show that this classification likely arises through the shared downstream biological effects of Hox family dysregulation. We also compare the NPM1c mutation signature to the *PML-RARA* signature and show a clear separation of these subtypes reflecting their divergent phenotypes and the mutually exclusive nature of these mutations.

While we demonstrate that singscore is capable of inferring mutation status from the transcriptional profile of AML samples, we note that it is best used to supplement alternative data which can provide a more definitive resolution of these lesions. Processing of raw RNA seq data will directly identify the presence of gene fusion products or mutations within protein-coding regions, although for many large data sets the quantified transcript abundance data are much easier to obtain without access agreements. The method can also be applied to legacy microarray data sets where genome and RNA sequencing data are unavailable. As such singscore provides a useful approach to supplement established methods for the study of genetic lesions in cancer. By exploring associations between different genomic and phenotypically relevant signatures, it may also help to better characterise true driver mutations which exert consistent effects on the transcriptome of AML samples and other cancers.

## Downloading and preparing the data

Data from TCGA project is made available through the Genomic Data Commons (GDC). Open access data from the project can be accessed in multiple pre-processed formats. Transcriptomic data can be downloaded either at the count level or as FPKM transformed abundance, before or after upper quantile normalisation. Other pre-processed version can be found from sources such as the cBioPortal and FireBrowse. The GDC data used STAR to perform a two-pass alignment followed by quantification using HTSeq. Data from the GDC can be downloaded using the GDC data transfer tool which allows users to select the specific files of interest using the GDC portal. These files then have to be read, merged, annotated and processed into a data structure that simplifies downstream analysis. Alternatively, all the above mentioned steps, including the download, can be performed using the R package TCGAbiolinks ^18^. The package supports data download using the GDC API and the GDC client. We will use the TCGAbiolinks package to download, annotate and process the data into a SummarizedExperiment R object.

The following steps need to be performed to prepare the data:

1. Create a query to select the files to download
2. Execute the query and download the data
3. Read the data into R
4. Filter out genes with low expression
5. Normalise the data for compositional bias and transform to account for gene-length biases as outlined in the singscore manuscript ^9^

### Querying the GDC database

The first step in any analysis should be to determine and report the data version and the service used to download the data. The getGDCInfo() function returns the release date of all data on the GDC along with a version.


library(SingscoreAMLMutations)
library(TCGAbiolinks)         

#get GDC version information  
gdc_info = getGDCInfo()       
gdc_info                      



## $commit
## [1] "b18b2385b1e916597856067dc6437f3c20b46bca"
##
## $data_release
## [1] "Data Release 19.0 - September 17, 2019"
##
## $status
## [1] "OK"
##
## $tag
## [1] "1.22.0"
##
## $version
## [1] 1


A query then needs to be run, using the GDC to identify the specific files for download. This step is similar to generating a *MANIFEST* file using the GDC portal. The first parameter of the query specifies the project - available projects can be accessed using getGDCprojects() or from https://portal.gdc.cancer.gov/projects. The TCGA acute myeloid leukemia data is part of the TCGA-LAML project. Following this, the data category, data type and workflow type need to be specified. The query formed below selects files containing the count level transcriptomic measurements. Values for different parameters of the query can be identified from “searching arguments” section of the “query” vignette: vignette("query", package = "TCGAbiolinks"). The result of this query will be a dataframe containing filenames and additional annotations related to the files.

Read count level data are selected instead of the processed FPKM data as one of the downstream pre-processing analysis results in filtering out of genes. A general recommendation is to compute FPKM values after filtering genes out so as to ensure counts are normalised by the corresponding library sizes. In cases where count-level data is not available, filtering can be performed directly on FPKM values, provided that the library size is large enough.


#form a query for the RNAseq data               
query_rna = GDCquery(                           
  #getGDCprojects()                             
  project = ’TCGA-LAML’,                        
#TCGAbiolinks:::getProjectSummary(’TCGA-LAML’)
data.category = ’Transcriptome Profiling’,    
data.type = ’Gene Expression Quantification’, 
workflow.type = ’HTSeq - Counts’              
)                                               
#extract results of the query                   
rnaseq_res = getResults(query_rna)              
dim(rnaseq_res)                                 



## [1] 151 28



colnames(rnaseq_res)                            



## [1] "data_release"               "data_type"
## [3] "updated_datetime"           "file_name"
## [5] "submitter_id"               "file_id"
## [7] "file_size"                  "cases"
## [9] "id"                         "created_datetime"
## [11] "md5sum"                    "data_format"
## [13] "access"                    "state"
## [15] "version"                   "data_category"
## [17] "type"                      "experimental_strategy"
## [19] "project"                   "analysis_id"
## [21] "analysis_updated_datetime" "analysis_created_datetime"
## [23] "analysis_submitter_id"     "analysis_state"
## [25] "analysis_workflow_link"    "analysis_workflow_type"
## [27] "analysis_workflow_version" "tissue.definition"


### Downloading the TCGA AML RNA-seq read counts

The GDCdownload function then executes the query on the GDC database and begins downloading the data using the GDC API. The download method should be changed to “client”, if the size of the data is expected to be large, e.g for RNA-seq read data or methylation data. It is good practice to specify the directory for data storage - we store all the data in the “GDCdata” directory in the temporary directory. Users should store their data in a permanent storage to retain the data. The function downloads the data and organises it into the folder based on parameters specified in the query. This allows multiple different levels and types of data to be stored in the same directory structure. Files with counts are stored at *TEMPDIR/GDCdata/TCGA-LAML/harmonized/Transcriptome_Profiling/Gene_Expression_Quantification/*.


datapath = file.path(tempdir(), ’GDCdata’)                
GDCdownload(query_rna, directory = datapath) #(size: 39MB)


### Reading count-level data into R

The GDCprepare function reads and processes the downloaded data into a RangedSummarizedExperiment object from the SummarizedExperiment package which allows patient annotations, gene annotations and count data to be stored in one object. Patient annotations are downloaded upon calling this function and subsequently mapped and attached to the resulting object. A RangedSummarizedExperiment object is similar to an ExpressionSet object but provides added functionality such as indexing with genomic coordinates and storing multiple data matrices with the same structure. Feature annotations used to annotate the data are stored in an RDA/RDATA file.


aml_se = GDCprepare(query_rna, directory = datapath)   


The object contains data for 56,925 features and 151 samples. The original data files contain 60,483 features, some of which (3,881) could not be mapped to ENSEMBL GRCh38.p12. Feature and sample annotations can be accessed using rowData(se) and colData(se), respectively, and the counts data can be accessed using assay(se). TCGA data usually contains some formalin-fixed paraffin-embedded (FFPE) samples which should be discarded from the analysis as the protocol introduces biological artefacts. This procedure is only performed on solid tumours and not leukemias, therefore, no filtering is required for this data set.


aml_se                                                 



## class: RangedSummarizedExperiment
## dim: 56512 151
## metadata(1): data_release
## assays(1): HTSeq - Counts
## rownames(56512): ENSG00000000003 ENSG00000000005 ...
##   ENSG00000281912 ENSG00000281920
## rowData names(3): ensembl_gene_id external_gene_name
##   original_ensembl_gene_id
## colnames(151): TCGA-AB-3001-03A-01T-0736-13
##   TCGA-AB-2853-03A-01T-0734-13 ... TCGA-AB-2977-03B-01T-0760-13
##   TCGA-AB-2995-03A-01T-0735-13
## colData names(61): sample patient ... name is_ffpe


### Filter out genes with low counts

The edgeR package contains methods that assist in the data normalisation and transformation required for filtering and subsequent steps. The methods require a DGEList object therefore we begin by creating a DGEList for the AML data from the SummarizedExperiment.


library(SummarizedExperiment)                                     
library(edgeR)                                                    

aml_dge = DGEList(counts = assay(aml_se), genes = rowData(aml_se))


**Figure 1.**
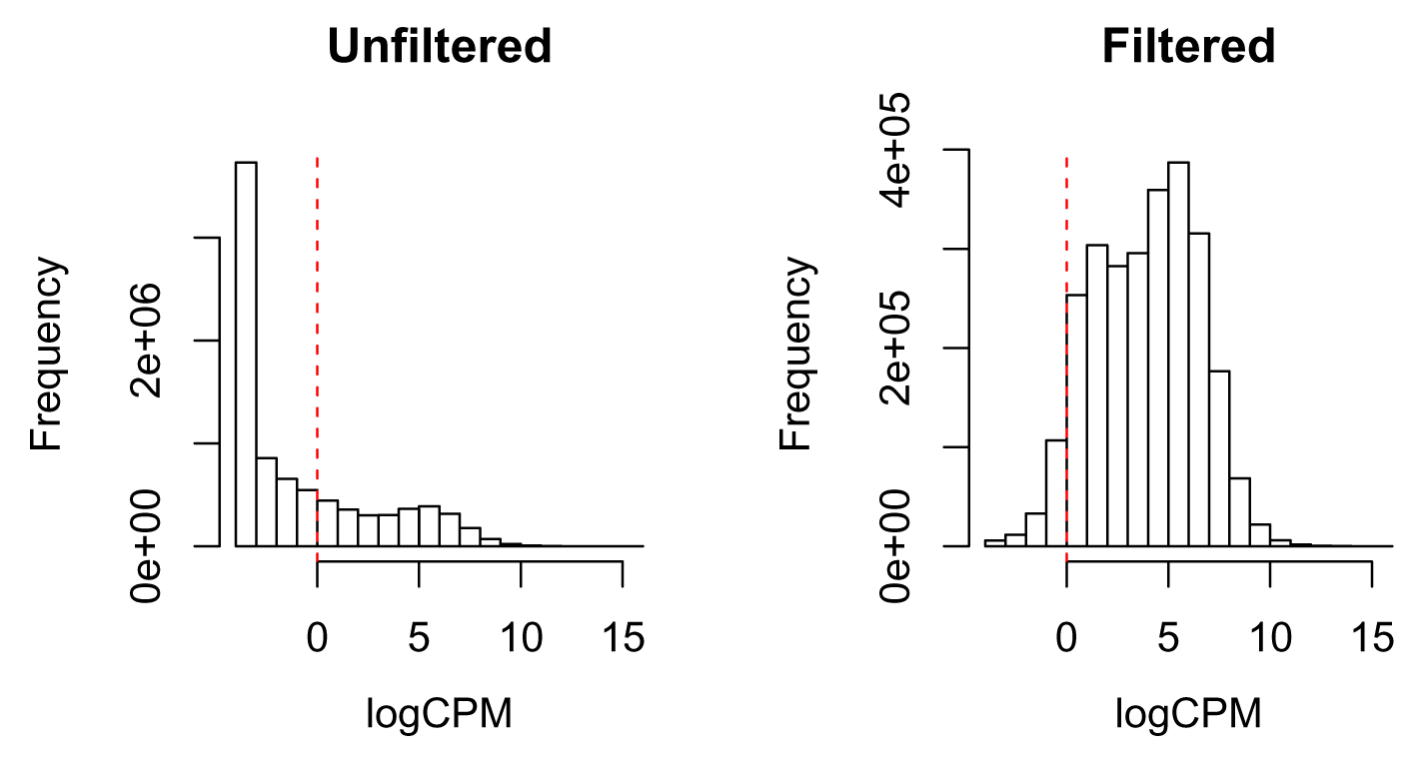
Histogram of logCPM values for the AML data before and after filtering. Filtering results in fewer zeros in the data. Most genes with CPM less than 1, logCPM < 0; (red line) across the majority of samples get discarded, resulting in an approximately log-normal distribution.


Genes with low counts across most samples are discarded from the analysis. This is a standard step in differential expression analysis as inclusion of such genes in the analysis could skew estimates of dispersion. It is also motivated in rank-based analysis, such as with singscore, to avoid rank duplication. Rank duplication reduces the discriminant power of scores as the number of unique ranks is reduced. A commonly used filter is to select only those genes that have CPMs above a certain threshold across a proportion of samples. Filtering is performed on the CPMs rather than raw counts as the former accounts for variation in library sizes, therefore, is unbiased. For instance, a CPM of 1 would equate to read counts between 19 and 50 for samples in the AML data where library sizes vary between 18.6 and 49.7 million reads. Here, we retain genes that have a CPM > 1 across more than 50% of the samples. Other methods to filter out genes with low counts exist and may be preferable in specific applications. Chen *et al*. ^19^ and Law *et al*. ^20^ filter genes based on the experimental design whereby the proportion of samples with enough read counts are evaluated per experimental group. As the AML data have many samples, filtering is performed across all samples rather than within sub-groups. Group specific filtering would be recommended for the study of rare groups. The distribution of logCPMs is much closer to the expected log-normal distribution after filtering out genes with low counts as seen in Figure 1.

prop_expressed = rowMeans(cpm(aml_dge) > 1)                                
keep = prop_expressed > 0.5                                                

op = par(no.readonly = TRUE)                                               
par(mfrow = c(1, 2))                                                       
hist(cpm(aml_dge, log = TRUE), main = ’Unfiltered’, xlab = ’logCPM’)       
abline(v = log(1), lty = 2, col = 2)                                       
hist(cpm(aml_dge[keep, ], log = TRUE), main = ’Filtered’, xlab = ’logCPM’) 
abline(v = log(1), lty = 2, col = 2)                                       



par(op)                             



#subset the data                                  
aml_dge = aml_dge[keep, , keep.lib.sizes = FALSE] 
aml_se = aml_se[keep, ]                           


### Transformation to FPKM values and normalisation

Singscore requires gene expression measurements to be comparable between genes within a sample, therefore, correction for gene length bias needs to be performed ^21^. Transformations such as transcripts per million (TPM) and reads/fragments per kilobase per million (RPKM/FPKM), that normalise by gene length, may be used. Both- TPM and RPKM/FPKM values should produce similar results when applying singscore provided that the library size is large enough, which they are here. RPKM values are generally computed after correcting for compositional biases. The calcNormFactors function in edgeR provides three methods to do so, TMM normalisation being the default. Chen *et al*. ^19^ and Law *et al*. ^20^ discuss the implications of normalisation prior to down-stream processing such as differential expression analysis. Normlisation is generally performed for cross-sample analysis where samples need to be comparable. Singscores are invariant to data normalisation as the analysis is contained within the sample of interest. The idea extends to any transformation that preserves the relative ranks of genes within a sample such as a log transformation. Here, we use TMM normalisation solely for visualisation purposes.

Data transformation to TPM or RPKM/FPKM requires the lengths for all genes to be calculated. Gene lengths need to be computed based on the alignment and quantification parameters. The TCGA transcriptomic data has been aligned using STAR and quantified using HTSeq (details of the pipeline available at https://docs.gdc.cancer.gov/Data/Bioinformatics_Pipelines/Expression_mRNA_Pipeline/). HTSeq quantifies reads mapping to the exons of each gene, therefore, effective gene lengths can be calculated as the sum of all exons spanning the gene. The GENCODE v22 annotation file was used for quantification therefore the same file needs to be used to compute gene lengths.


#download v22 of the GENCODE annotation                                     
gencode_file = ’gencode.v22.annotation.gtf.gz’                              
gencode_link = paste(                                                       
  ’ftp://ftp.ebi.ac.uk/pub/databases/gencode/Gencode_human/release_22’,     
  gencode_file,                                                             
  sep = ’/’                                                                 
  )                                                                         
download.file(gencode_link, gencode_file, method = ’libcurl’) #(size: 39MB) 


The rtracklayer R package provides functions to help parse GTF files.


library(rtracklayer)                                                                        
library(plyr)                                                                               

gtf = import.gff(gencode_file, format = ’gtf’, genome = ’GRCm38.71’, feature.type = ’exon’) 
#split records by gene to group exons of the same gene                                      
grl = reduce(split(gtf, elementMetadata(gtf)$gene_id))                                      
gene_lengths = ldply(grl, function(x) {                                                     
  #sum up the length of individual exons                                                    
    return(c(’gene_length’ = sum(width(x))))                                                
}, .id = ’ensembl_gene_id’)                                                                 


Genes are also annotated with their biotype for further analysis. The annotation file uses Ensembl IDs with versions as keys to records, which then need to be converted to Ensembl IDs. This is simply achieved by truncating the trailing version number.


#extract information on gene biotype                                               
genetype = unique(elementMetadata(gtf)[, c(’gene_id’, ’gene_type’)])               
colnames(genetype)[1] = ’ensembl_gene_id’                                          
gene_lengths = merge(genetype, gene_lengths)                                       

#remove ENSEMBL ID version numbers                                                 
gene_lengths$ensembl_gene_id = gsub(’\\.[0-9]*’, ’’, gene_lengths$ensembl_gene_id) 
saveRDS(gene_lengths, file = ’gene_lengths_HTSeq_gencodev22.rds’)                  
gene_lengths                                                                       



## DataFrame with 60483 rows and 3 columns
##       ensembl_gene_id      gene_type gene_length
##           <character>    <character>   <integer>
## 1     ENSG00000000003 protein_coding        4535
## 2     ENSG00000000005 protein_coding        1610
## 3     ENSG00000000419 protein_coding        1207
## 4     ENSG00000000457 protein_coding        6883
## 5     ENSG00000000460 protein_coding        5967
## ...               ...            ...         ...
## 60479 ENSGR0000275287       misc_RNA         290
## 60480 ENSGR0000276543          miRNA          68
## 60481 ENSGR0000277120          miRNA          64
## 60482 ENSGR0000280767        lincRNA         515
## 60483 ENSGR0000281849      antisense         484


The SummarizedExperiment object allows feature annotations to be stored, therefore, information on gene length and biotypes should be added to the existing annotations. Similarly, annotations need to be added to the DGEList object. The column containing lengths should include “length” in its name.


#allocate rownames for ease of indexing                                     
rownames(gene_lengths) = gene_lengths$ensembl_gene_id                       
rowData(aml_se)$gene_length = gene_lengths[rownames(aml_se), ’gene_length’] 
rowData(aml_se)$gene_biotype = gene_lengths[rownames(aml_se), ’gene_type’]  

#annotate gene lengths for the DGE object                                   
aml_dge$genes$length = gene_lengths[rownames(aml_dge), ’gene_length’]       


RPKM/FPKM values can now be calculated with the computed gene lengths after computing the normalisation factors. The SummarizedExperiment object can store multiple levels of the data simultaneously, provided that the number of features and samples remains the same across measurements. As such, FPKM values are appended to the existing object.


aml_dge_tmm = calcNormFactors(aml_dge, method = ’TMM’)       

#compute FPKM values and append to assays                    
assay(aml_se, ’logFPKM_TMM’) = rpkm(aml_dge_tmm, log = TRUE) 
aml_se                                                       



## class: RangedSummarizedExperiment
## dim: 17412 151
## metadata(1): data_release
## assays(2): HTSeq - Counts logFPKM_TMM
## rownames(17412): ENSG00000000419 ENSG00000000457 ...
##   ENSG00000281772 ENSG00000281896
## rowData names(5): ensembl_gene_id external_gene_name
##   original_ensembl_gene_id gene_length gene_biotype
## colnames(151): TCGA-AB-3001-03A-01T-0736-13
##   TCGA-AB-2853-03A-01T-0734-13 ... TCGA-AB-2977-03B-01T-0760-13
##   TCGA-AB-2995-03A-01T-0735-13
## colData names(61): sample patient ... name is_ffpe


### Annotate samples with mutation data

For this analysis we have used the curated mutation list from the original TCGA AML publication ^3^ (Supplemental Table 01 at https://gdc.cancer.gov/node/876) rather than variant calls from the standard TCGA pipeline (available through the National Cancer Institute Genomic Data Commons) and readers should note that there are some discrepancies between these. For genetic lesions of interest (NPM1c, *KMT2A-MLL*, *KMT2A*-PTD and *PML-RARA*), patients were identified by the following criteria:

- **Patient ID**: The ‘TCGA Patient ID’ column was extracted directly
- **NPM1c**: TRUE if the ‘NPM1’ column contains the strings ‘p.W287fs’ or ‘p.W288fs’
- **KMT2A-fusion**: TRUE if the ‘MLL-partner’ column contains the string ‘MLL-’ or ‘-MLL’ (note that the official gene symbol for *MLL* is now *KMT2A*)
- **KMT2A-PTD**: TRUE if the ‘MLL-PTD’ column contains the string ‘exons’
- **PML-RARA**: TRUE if the ‘PML-RARA’ column contains the string ‘PML-RARA’

data(AMLPatientMutationsTCGA)                                            
patient_mutations = AMLPatientMutationsTCGA                              
patient_mutations = patient_mutations[colnames(aml_se), ] # order samples
aml_mutations = colnames(patient_mutations) # get mutation labels        
colData(aml_se) = cbind(colData(aml_se), patient_mutations)              
colData(aml_se)[, aml_mutations]                                         



## DataFrame with 151 rows and 4 columns
##                              NPM1c.Mut KMT2A.Fusion KMT2A.PTD  PML.RARA
##                              <logical>    <logical> <logical> <logical>
## TCGA-AB-3001-03A-01T-0736-13     FALSE        FALSE     FALSE      TRUE
## TCGA-AB-2853-03A-01T-0734-13      TRUE        FALSE     FALSE     FALSE
## TCGA-AB-2929-03A-01T-0735-13     FALSE        FALSE     FALSE     FALSE
## TCGA-AB-2939-03A-01T-0740-13     FALSE        FALSE     FALSE     FALSE
## TCGA-AB-2982-03B-01T-0748-13     FALSE        FALSE     FALSE      TRUE
## ...                                ...          ...       ...       ...
## TCGA-AB-2815-03A-01T-0734-13     FALSE        FALSE     FALSE     FALSE
## TCGA-AB-3000-03A-01T-0736-13     FALSE        FALSE     FALSE     FALSE
## TCGA-AB-2919-03A-01T-0740-13      TRUE        FALSE     FALSE     FALSE
## TCGA-AB-2977-03B-01T-0760-13     FALSE        FALSE      TRUE     FALSE
## TCGA-AB-2995-03A-01T-0735-13     FALSE        FALSE     FALSE     FALSE


### Map Ensembl IDs to Entrez IDs

Ensembl annotations (Ensembl IDs) have higher coverage of the genome which may be useful in applications such as variant calling and similar exploratory analysis ^22^. However, RefSeq annotations (Entrez IDs) may be better suited to RNA-seq analyses which require a stable reference annotation for comparison ^23^. As such, we choose to map Ensembl IDs to Entrez IDs and discard any unmapped features.

Mapping can be performed using the Ensembl Biomart service, which can be queried using the biomaRt bioconductor package. This would provide the most up to date annotations. Alternatively, mapping could be performed with the bi-annually updated org.Hs.eg.db annotation R package which provides a stable set of annotations, thereby enhancing reproducibility. Mapping is performed with the mapIds function in the AnnotationDbi R package.


library(org.Hs.eg.db)               

rowData(aml_se)$entrezgene = mapIds(
  org.Hs.eg.db,                     
  keys = rownames(aml_se),          
  keytype = ’ENSEMBL’,              
  column = ’ENTREZID’,              
  multiVals = ’asNA’                
  )                                 
gene_annot = rowData(aml_se)        


Multimapped Ensembl IDs are replaced by NAs, then discarded to enforce unique mapping. Similarly, Entrez IDs that map to multiple Ensembl IDs are identified from the mapping, and discarded. Only features with unique Ensembl ID to Entrez ID mappings remain.


#select genes with mapped Entrez IDs                                           
keep = !is.na(gene_annot$entrezgene)                                           

#select genes with unique Entrez IDs                                           
dup_entrez = gene_annot$entrezgene[duplicated(gene_annot$entrezgene)]          
keep = keep & !gene_annot$entrezgene %in% dup_entrez                           

#Biotype of discarded genes (due to non-unique mapping)                        
head(sort(table(gene_annot[!keep, ’gene_biotype’]), decreasing = TRUE), n = 10)



##
##         processed_pseudogene                          antisense
##                         1017                                903
##                      lincRNA                                TEC
##                          451                                263
##               sense_intronic                     protein_coding
##                          249                                138
##       unprocessed_pseudogene transcribed_unprocessed_pseudogene
##                          128                                 93
##         processed_transcript   transcribed_processed_pseudogene
##                           72                                 72



#subset the data                                                               
aml_se = aml_se[keep, ]                                                        


## Transcriptional signatures to predict mutation status

The signature by Verhaak *et al*. ^12^ is now used to predict the mutation status of the NPM1c mutation. This is done by quantifying the concordance of genes in the signature with their expression in each sample. As such, high expression of up-regulated genes and low expression of down-regulated genes would result in higher scores. This single value can then be used to predict the mutation status of individual samples if these data were unavailable.

The signatures of interest are first downloaded from the MSigDB and read into GeneSet objects from the GSEABase R package. We then use the singscore R/Bioconductor package to quantify each sample for the Verhaak signature. Some of the visualisation and diagnostic tools within the singscore package are used to interpret the signatures and scores. Finally, we use a simple logistic regression model on the scores to predict the mutation status.

### Download signature and load into R

The Verhaak *et al*. ^12^ signature is composed of an up-regulated and a down-regulated gene set. Many signatures are developed in such a manner to improve discrimination of samples. MSigDB stores such signatures using names with suffixes "_UP" and "_DN" representing the independent components of the signature. Here, we form the download links for the signature with the base name “VERHAAK_AML_WITH_NPM1_MUTATED”.


#create signature names                                                          
verhaak_names = paste(’VERHAAK_AML_WITH_NPM1_MUTATED’, c(’UP’, ’DN’), sep = ’_’) 
verhaak_names                                                                    



## [1] "VERHAAK_AML_WITH_NPM1_MUTATED_UP" "VERHAAK_AML_WITH_NPM1_MUTATED_DN"


The signatures are then downloaded using the links, resulting in an XML file for each component of the signature. The mapply function is used to run the download function on all pairs of link-output arguments.


#generate URLs                                                                       
  verhaak_links = paste0(                                                              
  ’http://software.broadinstitute.org/gsea/msigdb/download_geneset.jsp?geneSetName=’,
verhaak_names,                                                                       
  ’&fileType=xml’                                                                    
  )                                                                                  

#download files                                                                      
verhaak_files = paste0(verhaak_names, ’.xml’)                                        
mapply(download.file, verhaak_links, verhaak_files, method = ’libcurl’)              


Functions in the GSEABase package help with reading, parsing and processing the signatures. Signatures from an MSigDB XML file can be read using the getBroadSets function which results in a GeneSet object. Gene symbols, Entrez IDs and affymetrix chip IDs from the original experiment (HG-U133A in this case) are stored in the XML file. Entrez IDs are read from the file as these can be mapped directly to our data. Conversions to other identifiers can be achieved using the mapIdentifiers function from GSEABase and an annotation package that contains the mappings. The advantage of using this function instead of the mapIds function from the AnnotationDbi package is that the former retains the GeneSet object after conversion of IDs.


library(GSEABase)                                                     

                            
verhaak_sigs = getBroadSets(verhaak_files, membersId = ’MEMBERS_EZID’)
verhaak_sigs                                                          



## GeneSetCollection
##   names: VERHAAK_AML_WITH_NPM1_MUTATED_UP, VERHAAK_AML_WITH_NPM1_MUTATED_DN (2 total)
##   unique identifiers: 10051, 10135, ..., 9828 (435 total)
##   types in collection:
##     geneIdType: SymbolIdentifier (1 total)
##     collectionType: BroadCollection (1 total)


To make data indexing easier during signature scoring, row names of the SummarisedExperiment object are changed to Entrez IDs which are already part of the row annotations.


rownames(aml_se) = rowData(aml_se)$entrezgene


### Score TCGA AML samples using the Verhaak signature

Singscore is a rank based metric of gene set enrichment in single samples. Scores for multiple signatures make use of the same ranked expression per sample. As such, it makes sense to compute the ranks only once and re-use them for scoring different signatures. The implementation separates these two phases of the analysis to reduce the computational cost of scoring. The rankGenes function will compute ranks from expression data in the form of either a numeric matrix, numeric data frame, ExpressionSet object, DGEList object or a SummarizedExperiment object. Users also have to specify what method should be used to break ties. The default is ‘min’ and we recommend this be used for RNA-seq data which may have many genes with zero counts. This will reduce the effect of zeros in the scores, however, appropriate pre-filtering of genes with low counts will still be required.


library(singscore)                                                          

#apply the rankGenes method to each version of the dataset, excluding counts
aml_ranked = rankGenes(assay(aml_se, ’logFPKM_TMM’))                        


Singscores can be computed using three modes, depending on the properties of the gene signature. The first mode of operation is applied when two directed gene sets (expected up- and down-regulated gene sets) form the transcriptomic signature. Many signatures in the MSigDB, including the Verhaak *et al*. ^12^ signature come in such pairs. This mode can be invoked by passing the up- and down-regulated gene sets to the arguments upSet and downSet respectively. In some cases, only one set of genes forms the signature. If all genes in the gene set are up-regulated or all down-regulated, the second mode of operation applies and is invoked by passing the gene set to the upSet argument. For sets of down-regulated genes, the score would simply be inverted (-score if scores are centered, 1 - score otherwise). Finally, if the user is unsure of the composition of genes in the gene-set, such that, the gene set may contain both up- and down- regulated genes, the final mode of singscore applies. The gene set is passed to the upSet argument similar to the previous mode with the additional argument knownDirection set to FALSE.

By default, singscores are centered such that the range of scores is [-1, 1] and [-0.5, 0.5] for the first two modes respectively. Negative scores indicate an inverse enrichment of signatures, that is, expected up-regulated genes are in fact down-regulated and vice-versa. Scores from the last mode can not be centered and have the range [0, 1]. In this mode, high scores are obtained when ranks of genes are distant from the median and low scores obtained when ranks converge to the median rank. If scores are centered in this scenario, it would lead to the conclusion that a negative score shows inverted enrichment, which is not the case. Score centering only serves the purpose of easing interpretation for users, a simple linear transformation is applied to achieve it.

Scores for the NPM1c mutation signature are computed using the default settings, with the first mode of operation being used due to the presence of an up- and down- regulated gene set. The function returns a data frame reporting the score and dispersion of ranks for the up-regulated gene set, down-regulated gene set and the combination of both. Dispersion of the combined gene set in this mode is simply the mean of the independent dispersion estimates. If any gene names/IDs are present in the signature but missing in the expression data, a warning will be reported.


#apply the scoring function                              
verhaak_scores = simpleScore(aml_ranked,                 
                             upSet = verhaak_sigs[[1]],  
                             downSet = verhaak_sigs[[2]])



## Warning in checkGenes(upSet, rownames(rankData)): 24 genes missing: 10265,
## 108, 10924, 11025, 11026, 1672, 200315, 2212, 2215, 3215, 3216, 3569, 3627,
## 3759, 50486, 6346, 6364, 6967, 8337, 861, 8843, 9518, 9627, 9997

## Warning in checkGenes(downSet, rownames(rankData)): 29 genes missing:
## 10232, 10267, 11217, 2122, 221981, 2258, 23532, 24141, 25907, 2697, 28526,
## 28638, 3047, 3386, 3848, 3934, 4070, 445, 4680, 4681, 5457, 5790, 6091,
## 653067, 653145, 7441, 8277, 862, 8788


It should be noted that singscores are composed of two components, an enrichment score and a dispersion estimate of ranks. The quantity of interest in gene set enrichment is the distribution of the expression or ranks of genes in the signature. In an ideal scenario, all expected up-regulated genes would have high expression therefore higher values of ranks. As such, ranks would be distributed on the higher end of the entire rank spectrum. Singscore aims to quantify this distribution of ranks, therefore, computes and reports the average and dispersion of ranks of genes in the signature relative to all other genes. The first quantity is similar to scores computed from all other single sample scoring methods. We determined a two component score to be a more appropriate and accurate representation of the distribution of ranks of signature genes. The default and recommended measure of dispersion is the median absolute deviation (MAD) due to its non-parametric property. Other appropriate measure of dispersion could be the inter-quartile range (IQR) and can be used by passing the IQR function as an argument to the dispersionFun argument.


head(verhaak_scores)



##                               TotalScore   TotalDispersion        UpScore
## TCGA-AB-3001-03A-01T-0736-13 -0.09161242          5256.929  -0.0768291807
## TCGA-AB-2853-03A-01T-0734-13  0.19605308          4882.943   0.0353047490
## TCGA-AB-2929-03A-01T-0735-13 -0.07106828          5443.737   0.0006426157
## TCGA-AB-2939-03A-01T-0740-13 -0.09795161          5697.632  -0.0599342558
## TCGA-AB-2982-03B-01T-0748-13 -0.11011396          5464.122  -0.0986913016
## TCGA-AB-2813-03A-01T-0736-13  0.15487900          4694.653   0.1385452964
##                              UpDispersion     DownScore  DownDispersion
## TCGA-AB-3001-03A-01T-0736-13      4513.034  -0.01478324        6000.823
## TCGA-AB-2853-03A-01T-0734-13      5868.872   0.16074833        3897.014
## TCGA-AB-2929-03A-01T-0735-13      5152.035  -0.07171089        5735.438
## TCGA-AB-2939-03A-01T-0740-13      4977.829  -0.03801735        6417.434
## TCGA-AB-2982-03B-01T-0748-13      5024.531  -0.01142266        5903.713
## TCGA-AB-2813-03A-01T-0736-13      4332.157   0.01633370        5057.149


### Diagnostics of the Verhaak signature

The singscore package provides a set of visualisation tools that enable diagnostics of the gene signature. For instance, these tools may be used to determine the importance of each component for a bidirectional signature (up- and down-regulated gene sets) to the total score, determine the importance of each gene of a signature in discriminating between the classes of interest, and to investigate the relationship between the final score and the dispersion of signature gene ranks. Sample annotations of interest (e.g. clinical annotations) can be colour coded on each plot. Singscore supports both continuous and categorical annotations, which can either be input as a vector, or as a string specifying a column within the score data frame. We begin by investigating the relationship between the score and dispersion of ranks for the up-regulated gene signature, down-regulated gene signature and the full signature. The plotDispersion functions generates a diagnostic plot with annotations overlaid. Annotations can be discrete or continuous, and can be passed as independent variables, or as a column name when the data is appended to the score data frame. It should be noted that all plotting functions in singscore can be made interactive by setting the isInteractive argument to TRUE.


#relative size of text in the figure                                         
relSize = 0.7                                                                

#create annotation                                                           
mutated_gene = rep(’Other’, ncol(aml_se))                                    
mutated_gene[aml_se$NPM1c.Mut] = ’NPM1c Mut’                                 
mutated_gene[aml_se$KMT2A.Fusion | aml_se$KMT2A.PTD] = ’MLL Fusion/PTD’      
p1 = plotDispersion(verhaak_scores, annot = mutated_gene, textSize = relSize)
p1


**Figure 2.**
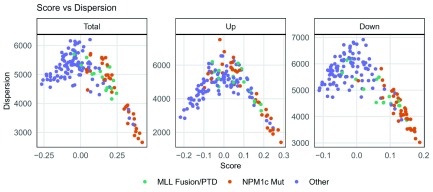
NPM1c signature scores from the up- and down-regulated gene sets, both combined (Total) and independently, can separate samples with NPM1c mutations or MLL fusions/PTDs from samples not bearing these genomic lesions. Scores are plotted against the median absolute deviation (MAD) of the ranks of genes in each gene set forming the NPM1c signature. The plots also reveal that scores close to 0 result in higher MADs.


Figure 2 shows that the set of down-regulated genes has more discriminant power in separating NPM1c mutated samples from those those without. Moreover, the signature is able to discriminate *MLL* ( *KMT2A*) fusions and PTDs from the other samples. NPM1c mutations produce higher scores for the down-regulated gene set while *MLL* fusions and PTDs produce moderate scores. Similar trends are observed with the set of upregulated genes; however, despite the range of scores increasing, the discriminant power drops moderately. In fact, the signature of up-regulated genes is able to discriminate samples without the genomic changes of interest (‘Other’) better by producing negative scores for most of these samples. Negative scores for the expected up-regulated gene set indicate that these genes are expressed below median expression, therefore, likely down-regulated within corresponding samples. Another observation from these plots is based on the trend of the dispersion (MAD). The dispersion is generally expected to be higher for scores close to zero. Zero scores are achieved in three possible scenarios: when genes are expressed at median expression level; when genes are evenly distributed at both ends of the expression spectrum; or, the more likely scenario whereby the rank of expression of all genes are uniformly distributed. The last two scenarios would result in a high dispersion. To explain these ideas, we select 3 samples and plot the ranks of genes in both signatures. The sample with the highest total score, lowest total score and highest dispersion are chosen.

library(gridExtra)                                                       

#select samples with the properties required                             
select_samples = c(                                                      
  ’Max Total Score’ = which.max(verhaak_scores$TotalScore),              
  ’Min Total Score’ = which.min(verhaak_scores$TotalScore),              
  ’Max Dispersion’ = which.max(verhaak_scores$TotalDispersion)           
  )                                                                      

#plotRankDensity applied to each sample                                  
rank_plots = lapply(names(select_samples), function(x) {                 
  #get the sample index                                                  
  aml_sample = select_samples[x]                                         
  #drop = FALSE is required to ensure the data.frame is intact           
  p1 = plotRankDensity(
                        rankData = aml_ranked[, aml_sample, drop = FALSE],
                       upSet = verhaak_sigs[[1]],                        
                       downSet = verhaak_sigs[[2]],                      
                       textSize = relSize)                               

 #overwrite title of the plot with the description of the sample         
 #this is possible because singscore uses ggplot2                        
 p1 = p1 + ggtitle(paste(x, mutated_gene[aml_sample], sep = ’\n’)) +     
   guides(colour = guide_legend(ncol = 1))                               

  return(p1)                                                             
})                                                                       

#create a multipanel plot                                                
grid.arrange(grobs = rank_plots, nrow = 1)                               


**Figure 3.**
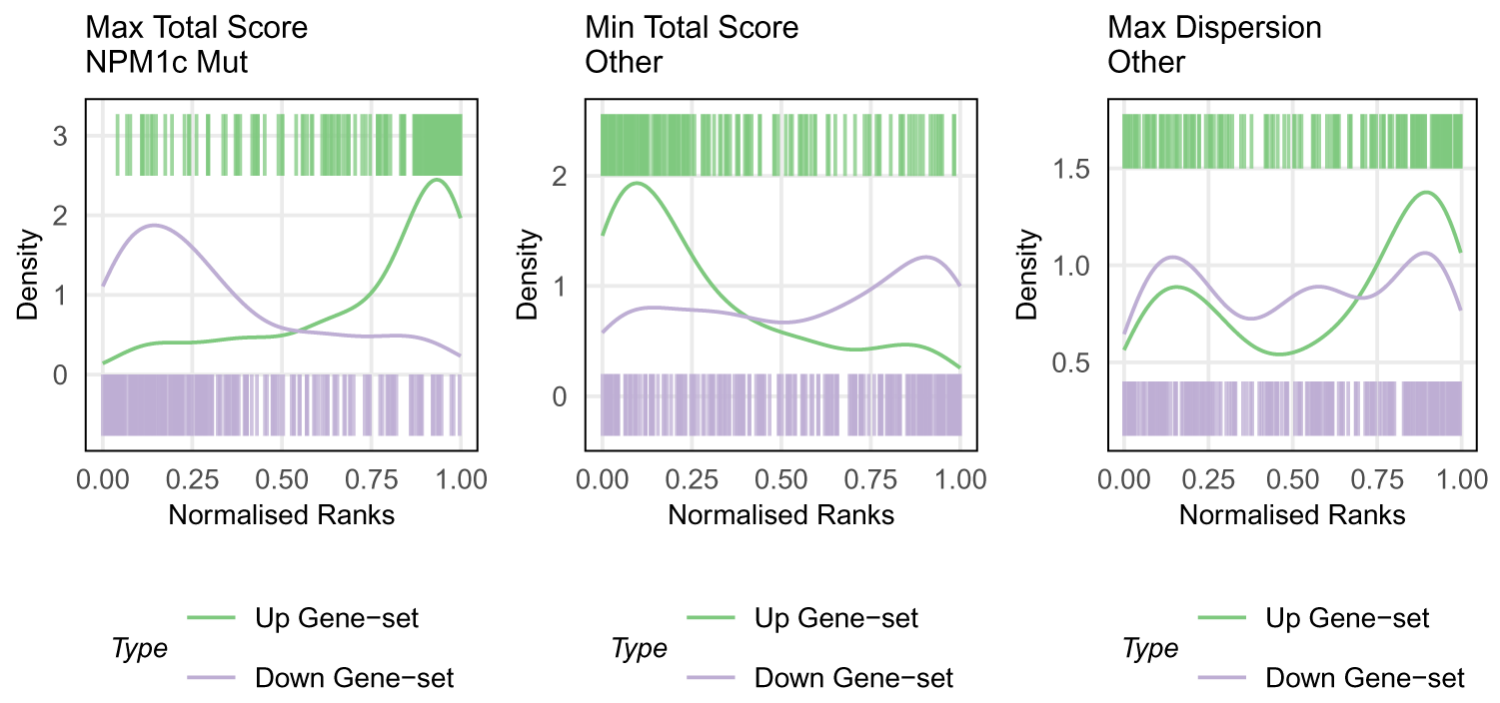
Rank distribution of genes reveals that expected up-regulated genes are up-regulated in high scoring samples and down-regulated in low-scoring samples with a similar, but inverse, observation for the expected down-regulated genes. Samples with the highest score, lowest score and highest dispersion (MAD) are show from left to right. The barcode plot for ranks of genes in each signature along with the density of the ranks is shown in each plot. Up-regulated genes are represented in green and down-regulated genes in purple. Gaussian distributions are observed at the extremes for the lowest and highest scores. High dispersion results in scores close to 0 because all genes are either evenly distributed (down-regulated gene set) or bi-modally distributed at the extrema (up-regulated gene set).

Figure 3 shows that both the up- and down-regulated gene sets contribute to the score of the top scoring sample as these genes are at the extremes ends of the rank spectrum. Similarly, the lowest scoring sample has the ranks of genes in each set inverted. As observed in the previous plot, the up-regulated genes improve discrimination between NPM1c mutated samples and other samples and may be a stronger indicator of wild-type NPM1c samples than the down-regulated genes. Finally, the sample with the maximum dispersion exhibits uniformly distributed down-regulated gene ranks and a bimodal distribution of the up-regulated gene ranks with the modes at the extremes of the spectrum.

Combined, these plots show that both the up- and down-regulated genes play an important role in discriminating between NPM1c mutated (and *MLL* fusion/PTD samples), and wild-type samples. These diagnostic plots help in determining the importance of genes in signatures with respect to the samples of interest and should be used prior to application of signatures. In some cases, signatures may have been developed in specific contexts due to inherent biases in datasets and yet, described or applied in a generalised setting. These diagnostic plots may help in validating their application in specific contexts and possibly assist in characterising the contexts of all existing signatures.

### Predicting NPM1c mutation status using the Verhaak signature

Mutation status can be predicted from singscores using a logistic regression model with a “logit” link function. The benefits of this model over one where each gene in the signature is used as a term in the model is the simplicity of the model. The Verhaak *et al*. ^12^ signature consists of 429 genes which would result in a regression model with 429 predictors. As there are fewer samples than the predictors, some feature selection method would be required which may result in the loss of information. Moreover, automating model development on a larger selection of gene signatures would be limited with gene-level models. Additionally, models trained using singscore would inherit its non-parametric properties to some extent. For instance, such models would be invariant to all data transformations that retain within sample ranks of genes. The main limitation of such models would be the loss of accuracy due to summarisation of the data (information on 429 genes is summarised into a two-component score).

In any case, our aim here is not to discuss the various models that can be used to predict mutation status, but to exhibit the discriminant power of singscore and transcriptomic signatures. consequently, the data used to train the model are used to evaluate the basic performance properties. We begin by combining the scores with mutation annotations.


#create a dataframe with the data required: scores and mutations 
scoredf = as.data.frame(colData(aml_se)[, aml_mutations])        
scoredf$Score = verhaak_scores$TotalScore                        
scoredf$Dispersion = verhaak_scores$TotalDispersion              


**Figure 4.**
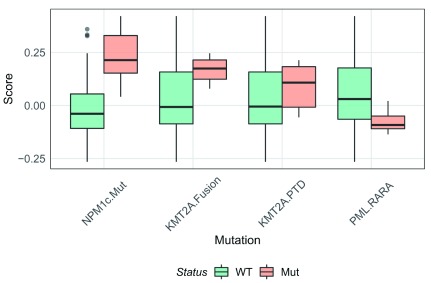
NPM1c signature scores are able to separate mutants from wild-types. Boxplot of scores from the NPM1c signature split by different types of mutations.


Before training a model, we can visualise how well scores resulting from the Verhaak *et al*. ^12^ signature separates the mutants from wild-type samples for some genes of interest. Figure 4 shows that NPM1c signature scores can discriminate between NPM1c wild-type and mutants. Similar observations are made for *MLL* ( *KMT2A*) fusions and *PML-RARA* fusions.

#restructure the data for ploting                             
plotdf = melt(                                                
  scoredf,                                                    
  id.var = c(’Score’, ’Dispersion’),                          
  variable.name = ’Mutation’,                                 
  value.name = ’Status’                                       
  )                                                           
#convert TRUE-FALSE values to Mut-WT                          
plotdf$Status = factor(plotdf$Status, labels = c(’WT’, ’Mut’))
p1 = ggplot(plotdf, aes(Mutation, Score, fill = Status)) +    
  geom_boxplot(position = ’dodge’, alpha = 0.6) +             
  scale_fill_brewer(palette = ’Set2’) +                       
  current_theme +                                             
  theme(axis.text.x = element_text(angle = 45, vjust = 0.5))  
p1                                                            



To quantify the above observations, we fit logistic regression models with each genomic lesion as the response variable and the score as the predictor. Coefficient estimates, standard errors, z-statistics, and *p*-values of each model are listed below.


#fit GLMs to each mutation                                           
glms = lapply(aml_mutations, function(x) {                           
  #generate a formula for the fit                                    
  form = as.formula(paste0(x, ’ ~ Score’))                           
  glm1 = glm(form, data = scoredf, family = binomial(link = ’logit’))
  return(glm1)                                                       
})                                                                   
names(glms) = aml_mutations                                          

#extract coefficients                                                
coefs = lapply(glms, function(x) coef(summary(x)))                   
ldply(coefs, function(x) x[2, ], .id = ’Mutation’)                   



##       Mutation  Estimate Std. Error    z value     Pr(>|z|)
## 1    NPM1c.Mut 13.276945   2.209811  6.0081806 1.876167e-09
## 2 KMT2A.Fusion  4.442768   2.151409  2.0650505 3.891822e-02
## 3    KMT2A.PTD  1.646183   2.089950  0.7876661 4.308921e-01
## 4     PML.RARA -6.780992   2.414752 -2.8081526 4.982661e-03


NPM1c mutations are significantly associated with the score. Samples carrying a *MLL* gene fusion are also associated with the score reflecting their shared effects on Hox gene dysregulation, although with a lower significance. Interestingly the *PML-RARA* fusion carries a negative coefficient, likely reflecting the distinct cellular morphology/phenotype of acute promyelocytic leukemia relative to other subsets of AML, as noted above.

### Evaluate prediction model performance

The above statistics give insight on the models trained but not on their performance. Precision and recall provide insight on a models predictive performance. The dcanr package provides functions to compute these metrics along with other measures of performance.


library(dcanr)                                                                 

#assess sensitivity and specificity                                            
prec_rec = ldply(glms, function(glm1) {                                        
  #predict mutations for the data used in training and convert to binary format
  prediction = as.numeric(predict(glm1) > 0)                                   
  observed = glm1$y                                                            
  prec = performanceMeasure(prediction, observed, ’precision’)                 
  recall = performanceMeasure(prediction, observed, ’recall’)                  
  f1 = performanceMeasure(prediction, observed)                                
  return(c(’Precision’ = prec, ’Recall’ = recall, ’F1’ = f1))                  
}, .id = ’Mutation’)                                                           
prec_rec                                                                       



##       Mutation Precision    Recall        F1
## 1    NPM1c.Mut       0.7 0.5675676 0.6268657
## 2 KMT2A.Fusion       NaN 0.0000000 0.0000000
## 3    KMT2A.PTD       NaN 0.0000000 0.0000000
## 4     PML.RARA       NaN 0.0000000 0.0000000



Precision for predictions of all genomic changes other than NPM1c is undefined because all samples are predicted to be wild-types. Consequently, their recall is zero. Precision, recall and the F1 score of the NPM1c mutation model are high as expected. As singscores are two-components scores, performance may be further improved by including the dispersion of ranks. This is observed in Figure 2.

As observed from the model below, using both components of singscores significantly improves the predictive performance.


#include dispersion in the model                                      
glm_npm1c = glm(’NPM1c.Mut ~ Score + Dispersion’,                     
               data = scoredf,                                        
               family = binomial(link = ’logit’))                     

#evaluate performance of the new model                                
prediction = as.numeric(predict(glm_npm1c) > 0)                       
observed = glm_npm1c$y                                                
c(                                                                    
  ’Precision’ = performanceMeasure(prediction, observed, ’precision’),
  ’Recall’ = performanceMeasure(prediction, observed, ’recall’),      
  ’F1’ = performanceMeasure(prediction, observed)                     
  )                                                                   



## Precision    Recall        F1
## 0.7631579 0.7837838 0.7733333


### Unsupervised classification of mutations

There may be some cases where sample annotation is not available. In such scenarios, we are unable to build regression models to interpret scores. A higher singscore would provide stronger evidence for the signature but the magnitude is difficult to interpret without a reference. One approach to deal with this situation is to compare scores to those from other datasets where the mutations status is known. An alternative approach would be to compare scores within the dataset using unsupervised learning methods.

Here we demonstrate the use of three clustering methods (Gaussian mixture decomposition, k-means clustering, and hierarchical clustering) to stratify samples, and as we have done previously ^24^ use the adjusted Rand index (ARI) to compare classifications. As expected, supervised (GLM) classification results in the best prediction. This is followed by clustering based on the score using Gaussian mixture decomposition. Any other classification algorithm along with prior knowledge could be used to decompose scores into groups. The important characteristic of singscores is that they maintain the discriminant power of gene signatures therefore can be coupled with supervised, semi-supervised or unsupervised algorithms to perform stratification.


library(mclust)                                                   

#Gaussian mixture model                                           
m1 = Mclust(scoredf$Score, G = 2, verbose = FALSE)                
#k-means clustering                                               
m2 = kmeans(scoredf[, 5:6], centers = 2, nstart = 100)            
#hierarchical clustering                                          
m3 = hclust(dist(scoredf[, 5:6]))                                 

mutation_inference = cbind(                                       
  ’GLM’ = prediction,                                             
  ’mclust’ = m1$classification,                                   
  ’k-means’ = m2$cluster,                                         
  ’hclust’ = cutree(m3, k = 2)                                    
)                                                                 
apply(mutation_inference, 2, adjustedRandIndex, scoredf$NPM1c.Mut)



##       GLM    mclust   k-means    hclust
## 0.5712724 0.4451106 0.3298026 0.3725541


## Signature landscapes with multiple signatures

Often, we are interested in the relationship between two dependent or independent phenotypes, for instance, the epithelial and mesenchymal phenotypes. The role of most signatures is to estimate an unobservable molecular phenotype so they may be considered as proxies of phenotypes. As such, we could investigate the relationship between two phenotypes using corresponding signatures. Foroutan *et al*. ^25^ first introduced the idea of molecular signature landscapes to investigate the relationship between signatures related to the epithelial-mesenchymal transition (EMT) and TGF *β*-induced EMT. Subsequently, Cursons *et al*. ^26^ computed epithelial and mesenchymal phenotype signature singscores and demonstrated an epithelial phenotype shift following miR-200c transfection into a mesenchymal cell line using a signature landscape. Foroutan *et al*. ^9^ used signature landscapes to stratify breast cancer subtypes along the epithelial-mesenchymal axis and included it as part of the singscore package. Here, we show how such landscapes can be used beyond the current application of the epithelial-mesenchymal axis. We demonstrate how transcriptomic signatures of different mutations can be used to stratify AML samples.

### Ross MLL fusion signature vs. Verhaak signature landscape

We now use the Ross *et al*. ^14^ MLL-fusion signatures to score the TCGA AML samples. Unlike the NPM1c signature, this signature is composed of genes that discriminate samples with MLL-fusion genes. We download and parse the signature as demonstrated with the NPM1c signature.


#create signature names                                                              
rossmll_name = ’ROSS_AML_WITH_MLL_FUSIONS’                                           
#generate URLs                                                                       
rossmll_link = paste0(                                                               
  ’http://software.broadinstitute.org/gsea/msigdb/download_geneset.jsp?geneSetName=’,
  rossmll_name,                                                                      
  ’&fileType=xml’                                                                    
  )                                                                                  

#download files                                                                      
rossmll_file = paste0(rossmll_name, ’.xml’)                                          
download.file(rossmll_link, rossmll_file, method = ’libcurl’)                        
rossmll_sig = getBroadSets(rossmll_file, membersId = ’MEMBERS_EZID’)                 
rossmll_sig                                                                          



## GeneSetCollection
##   names: ROSS_AML_WITH_MLL_FUSIONS (1 total)
##   unique identifiers: 10113, 10479, ..., 9961 (81 total)
##   types in collection:
##     geneIdType: SymbolIdentifier (1 total)
##     collectionType: BroadCollection (1 total)


The gene set is a composition of both up- and down-regulated genes as genes were selected based on their ability to discriminate mutants from wild-types. We use the third mode of singscore, which does not require the direction of genes in the gene set to be known. The knownDirection parameter of the simpleScore function is set to FALSE to induce this mode. Ranks computed previously can be reused to compute scores with the new signature.


rossmll_scores = simpleScore(aml_ranked, rossmll_sig[[1]], knownDirection = FALSE)


The plotScoreLandscape function plots a hexbin plot to visualise the two-dimensional distribution of scores. Scores computed using the Verhaak *et al*. ^12^ signature and the Ross *et al*. ^14^ *MLL*-fusions signature are passed as arguments. Both scores should have been computed on the same samples with the order of samples retained. Names of the scores should be passed as arguments to the scorenames argument. The textSize argument can be used to specify the size of all text relative to the plot size. This may prove useful when plots are being generated for scientific posters, publications and presentations, all of which require different image sizes.


p_mll_npm1c = plotScoreLandscape(                                              
  verhaak_scores,                                                              
  rossmll_scores,                                                              
  scorenames = c(’VERHAAK_AML_WITH_NPM1_MUTATED’, ’ROSS_AML_WITH_MLL_FUSIONS’),
  textSize = relSize                                                           
  )                                                                            
p_mll_npm1c                                                                    


**Figure 5.**
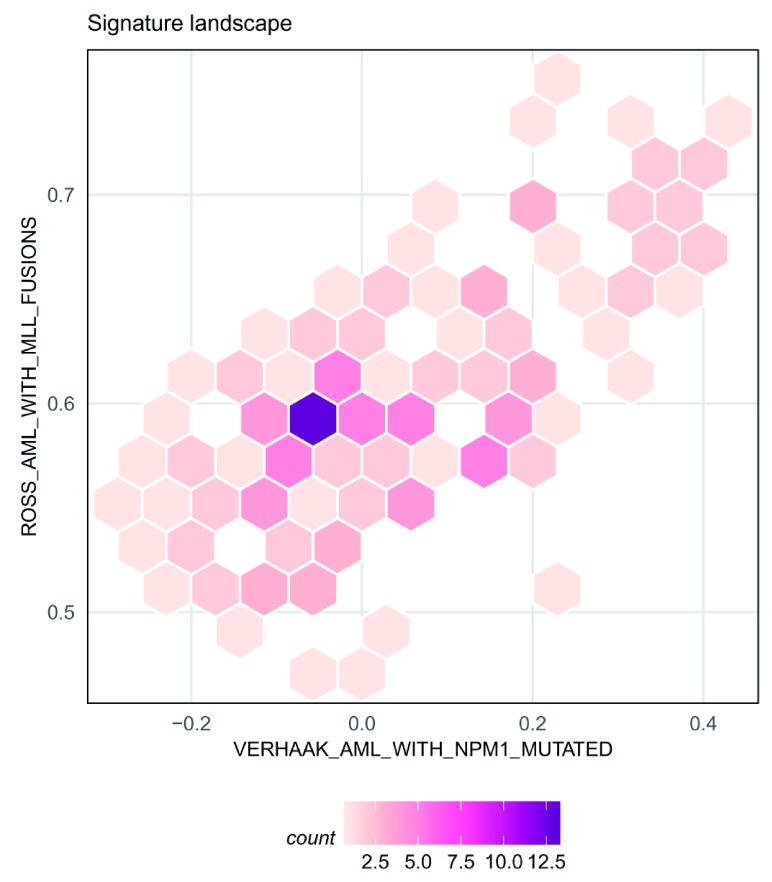
Signature landscape between the MLL fusion and NPM1c signatures. A positive association is revealed between the two signatures in AML.


Figure 5 shows a strong positive association between scores from the two signatures (Spearman’s *ρ* = 0.628) despite only 16 genes being shared across the two signatures (signature sizes are 78 and 429 genes). In such an analysis, we may be interested in projecting new data points onto the landscape as done by Cursons *et al*. ^26^. Alternatively, we may want to overlay some existing data points to investigate sample stratification using scores. Here, we overlay *MLL* fusions, *MLL* PTDs, *PML-RARA* fusions and NPM1c mutations onto the landscape. First, we build the annotation vector.

#new annotation - modify previously used annotations
mutated_gene[aml_se$KMT2A.Fusion] = ’MLL Fusion’    
mutated_gene[aml_se$KMT2A.PTD] = ’MLL PTD’          
mutated_gene[aml_se$PML.RARA] = ’PML-RARA’          



Points are projected onto an existing landscape using the projectScoreLandscape function. This functions uses the p_mll_npm1c plot generated using the plotScoreLandscape and overlays new data points onto it. Scores for the new data must be computed with the same signatures that were used to compute the landscape. As we are using existing data, scores computed in earlier sections are re-used. The subSamples can be used to select a subset of samples to project. Here, we select samples with the mutations we are interested in annotating.


select_aml = !mutated_gene %in% ’Other’                                         

#label samples with an mclust NPM1c classification uncertainty of > 0.3         
label_samples = substr(rownames(verhaak_scores), 6, 12) #sample ID from barcodes
label_samples[m1$uncertainty < 0.3] = NA                                        

#project mutations onto the landscape                                     
p1 = projectScoreLandscape(                                                     
  p_mll_npm1c,                                                                  
  verhaak_scores,                                                               
  rossmll_scores,                                                               
  subSamples = select_aml,                                                      
  annot = mutated_gene[select_aml],                                             
  sampleLabels = label_samples[select_aml]                                      
)                                                                               
p1 + theme(legend.box = ’vertical’)                                             


**Figure 6.**
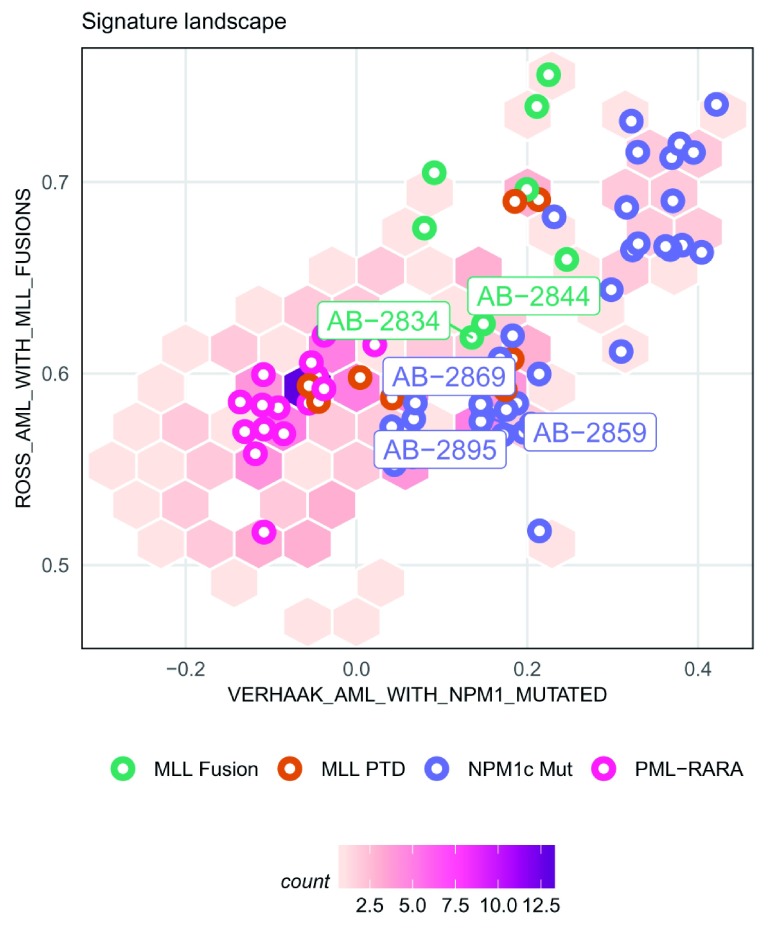
The landscape reveals a distinction between the MLL fusions and PTDs as previously reported. Different mutations occupy different regions of the landscape, possessing different types of molecular traits.

Figure 6 shows that the *MLL* fusions and *MLL* PTDs exhibit variation across different axes. *MLL* PTDs have a lower score than MLL fusions for the *MLL* fusion signature as expected. These sets of samples do not cluster along the two axes, consistent with observations by Ross *et al*. ^14^. Overlaying samples annotations onto the plot assists in interpreting different regions of the landscape.

### Ross PML-RARA fusion signature vs. Verhaak signature landscape

It is evident from the previous analysis that *PML-RARA* samples differ from all other samples examined here. We repeat the analysis with the *PML-RARA* signature from Ross *et al*. ^14^ to verify this distinction. The signature is available on the MSigDB and is named “ROSS_AML_WITH_PML_RARA_FUSION”. We download and parse the signature, score all samples against it, plot a landscape with scores from the Verhaak *et al*. ^12^ signature, and finally, project samples onto the plot. The signature was constructed in a similar manner to the *MLL* fusions signature, therefore, samples are scored using the same settings.

**Figure 7.**
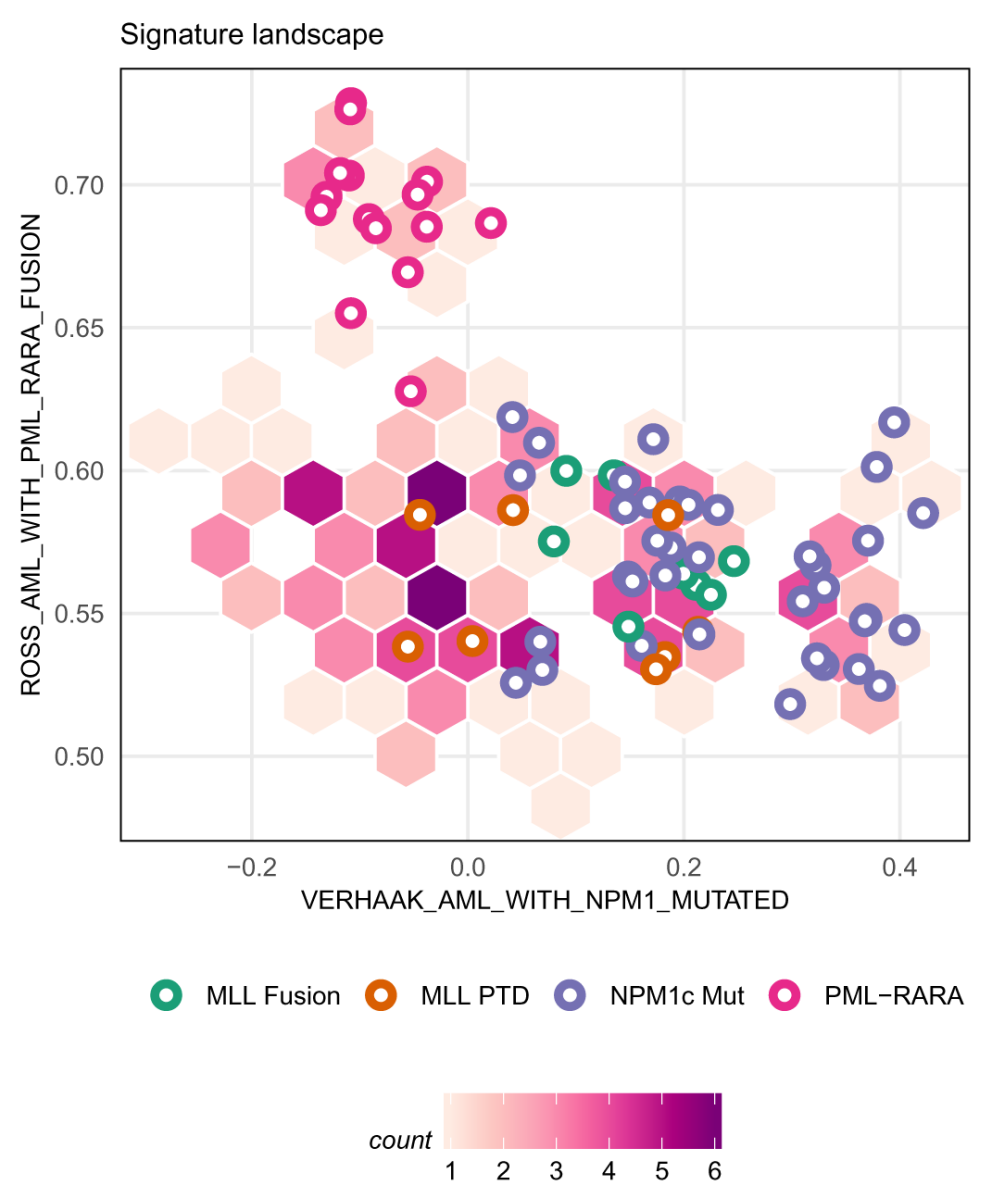
The PML-RARA signature is independent from the NPM1c signature in AML. The L-shaped landscape suggests the molecular mechanisms underlying these mutations are mutually exclusive.

Figure 7 shows a completely different landscape from what was observed with the *MLL* fusion signature. The *PML-RARA* signature forms a clear separation between the *PML-RARA* and NPM1c samples such that *PML-RARA* samples are the only ones with a high score for this signature. Moreover, no association is observed between the two signatures. As discussed above (Description of the biological problem) the *PML-RARA* fusion is diagnostic of a specific subtype of AML known as acute promyelocytic leukemia, with a highly distinct cell phenotype reflecting a block on differentiation at the promyelocyte stage ^15^. The distinct features of this subtype are correspondingly reflected in the L-shaped landscape for these two signatures and the different mechanisms by which these lesions drive leukemogenesis.

## Summary

The singscore package provides an easy interface to apply gene set scoring methods within the R/Bioconductor environment. The TCGAbiolinks package allows relatively easy access to large clinically relevant data sets such as TCGA, together with appropriate annotation functions for interpreting biological data. Diagnostic and plotting functions included with singscore allow the user to investigate gene sets of interest to determine their power for distinguishing differences between samples. Different gene signatures can then be combined to explore how different cellular phenotypes are associated across a large cohort of cancer samples. As demonstrated, when appropriate gene set signatures are used, metrics calculated by singscore can be used for sample classification and this may be useful for further interrogation of large transcriptomic data sets where no genomic data are available

## Packages used

This workflow depends on various packages from version 3.9 of the Bioconductor project, running on R version 3.6.1 (2019-07-05) or higher. The complete list of the packages used for this workflow are shown below:


sessionInfo()                                                              



## R version 3.6.1 (2019-07-05)
## Platform: x86_64-pc-linux-gnu (64-bit)
## Running under: CentOS Linux 7 (Core)
##
## Matrix products: default
## BLAS:   /stornext/System/data/apps/R/R-3.6.1/lib64/R/lib/libRblas.so
## LAPACK: /stornext/System/data/apps/R/R-3.6.1/lib64/R/lib/libRlapack.so
##
## locale:
##  [1] LC_CTYPE=en_US.UTF-8       LC_NUMERIC=C
##  [3] LC_TIME=en_US.UTF-8        LC_COLLATE=en_US.UTF-8
##  [5] LC_MONETARY=en_US.UTF-8    LC_MESSAGES=en_US.UTF-8
##  [7] LC_PAPER=en_US.UTF-8       LC_NAME=C
##  [9] LC_ADDRESS=C               LC_TELEPHONE=C
## [11] LC_MEASUREMENT=en_US.UTF-8 LC_IDENTIFICATION=C
##
## attached base packages:
## [1] parallel  stats4    stats     graphics  grDevices utils     datasets
## [8] methods   base
##
## other attached packages:
##  [1] mclust_5.4.5               dcanr_1.1.4
##  [3] gridExtra_2.3              reshape2_1.4.3
##  [5] singscore_1.4.0            GSEABase_1.46.0
##  [7] graph_1.62.0               annotate_1.62.0
##  [9] XML_3.98-1.20              org.Hs.eg.db_3.8.2
## [11] AnnotationDbi_1.46.0       plyr_1.8.4
## [13] rtracklayer_1.44.0         edgeR_3.26.5
## [15] limma_3.40.2               SummarizedExperiment_1.14.1
## [17] DelayedArray_0.10.0        BiocParallel_1.18.1
## [19] matrixStats_0.55.0         Biobase_2.44.0
## [21] GenomicRanges_1.36.1       GenomeInfoDb_1.20.0
## [23] IRanges_2.18.2             S4Vectors_0.22.1
## [25] BiocGenerics_0.30.0        TCGAbiolinks_2.12.3
## [27] ggplot2_3.2.0              SingscoreAMLMutations_1.0.1            
##
## loaded via a namespace (and not attached):
##   [1] backports_1.1.4            circlize_0.4.6
##   [3] aroma.light_3.14.0         igraph_1.2.4.1
##   [5] selectr_0.4-1              ConsensusClusterPlus_1.48.0
##   [7] lazyeval_0.2.2             splines_3.6.1
##   [9] usethis_1.5.1              sva_3.32.1
##  [11] digest_0.6.20              foreach_1.4.4
##  [13] htmltools_0.3.6            magrittr_1.5
##  [15] memoise_1.1.0              BSgenome_1.52.0
##  [17] cluster_2.1.0              doParallel_1.0.14
##  [19] ComplexHeatmap_2.0.0       Biostrings_2.52.0
##  [21] readr_1.3.1                R.utils_2.9.0
##  [23] prettyunits_1.0.2          colorspace_1.4-1
##  [25] blob_1.1.1                 rvest_0.3.4
##  [27] ggrepel_0.8.1              BiocWorkflowTools_1.10.0
##  [29] xfun_0.8                   dplyr_0.8.3
##  [31] hexbin_1.27.3              crayon_1.3.4
##  [33] RCurl_1.95-4.12            jsonlite_1.6     
##  [35] genefilter_1.66.0          survival_2.44-1.1
##  [37] zoo_1.8-6                  iterators_1.0.10
##  [39] glue_1.3.1                 survminer_0.4.4
##  [41] registry_0.5-1             gtable_0.3.0
##  [43] zlibbioc_1.30.0            XVector_0.24.0
##  [45] GetoptLong_0.1.7           shape_1.4.4
##  [47] scales_1.0.0               DESeq_1.36.0
##  [49] rngtools_1.4               DBI_1.0.0
##  [51] bibtex_0.4.2               ggthemes_4.2.0
##  [53] Rcpp_1.0.2                 xtable_1.8-4
##  [55] progress_1.2.2             cmprsk_2.2-8
##  [57] clue_0.3-57                bit_1.1-14
##  [59] matlab_1.0.2               km.ci_0.5-2
##  [61] httr_1.4.0                 RColorBrewer_1.1-2
##  [63] pkgconfig_2.0.2            R.methodsS3_1.7.1
##  [65] locfit_1.5-9.1             labeling_0.3
##  [67] tidyselect_0.2.5           rlang_0.4.0
##  [69] munsell_0.5.0              tools_3.6.1
##  [71] downloader_0.4             generics_0.0.2
##  [73] RSQLite_2.1.1              broom_0.5.2
##  [75] evaluate_0.14              stringr_1.4.0
##  [77] yaml_2.2.0                 knitr_1.23
##  [79] bit64_0.9-7                fs_1.3.1
##  [81] survMisc_0.5.5             purrr_0.3.2
##  [83] doRNG_1.7.1                EDASeq_2.18.0
##  [85] nlme_3.1-140               R.oo_1.22.0
##  [87] xml2_1.2.0                 biomaRt_2.40.1
##  [89] compiler_3.6.1             rstudioapi_0.10
##  [91] curl_3.3                   png_0.1-7
##  [93] ggsignif_0.5.0             tibble_2.1.3
##  [95] geneplotter_1.62.0         stringi_1.4.3
##  [97] GenomicFeatures_1.36.3     lattice_0.20-38
##  [99] Matrix_1.2-17              KMsurv_0.1-5
## [101] pillar_1.4.2               BiocManager_1.30.4 
## [103] GlobalOptions_0.1.0        data.table_1.12.2
## [105] bitops_1.0-6               R6_2.4.0
## [107] latticeExtra_0.6-28        hwriter_1.3.2
## [109] bookdown_0.11              ShortRead_1.42.0
## [111] codetools_0.2-16           assertthat_0.2.1
## [113] pkgmaker_0.27              rjson_0.2.20
## [115] withr_2.1.2                GenomicAlignments_1.20.1
## [117] Rsamtools_2.0.0            GenomeInfoDbData_1.2.1
## [119] mgcv_1.8-28                hms_0.4.2
## [121] grid_3.6.1                 tidyr_0.8.3
## [123] rmarkdown_1.13             git2r_0.26.1
## [125] ggpubr_0.2.1


## Data availability

All data analyzed in the workflow is read automatically from public websites as part of the code. Mutation data for the samples in this study are available as part of the R/Bioconductor package SingscoreAMLMutations (v1.0.0).

## Software availability

**Software available from:** https://bioconductor.org/packages/release/workflows/html/SingscoreAMLMutations.html

**Source code available from:** https://github.com/DavisLaboratory/SingscoreAMLMutations/

**Archived source code at time of publication:** https://doi.org/10.5281/zenodo.3470443

**License:** Artistic-2.0

## Author information

DDB, JC and MJD wrote the article. DDB wrote the code for the workflow and the associated R/Bioconductor workflow package SingscoreAMLMutations. MF, JC, DDB and MJD conceived the idea for the workflow. YX translated the vignette for the package vignette into chinese. YX and RL reviewed the chinese translation. All authors have tested and reviewed the workflow. This work was supervised by JC and MJD.

## References

[1] Cieślik M Chinnaiyan AM: Cancer transcriptome profiling at the juncture of clinical translation. *Nat Rev Genet.* 2018;19(2):93–109. 10.1038/nrg.2017.96 29279605

[2] Parker JS Mullins M Cheang MC: Supervised risk predictor of breast cancer based on intrinsic subtypes. *J Clin Oncol.* 2009;27(8):1160–7. 10.1200/JCO.2008.18.1370 19204204 PMC2667820

[3] Cancer Genome Atlas Research Network Ley TJ Miller C: Genomic and epigenomic landscapes of adult *de novo* acute myeloid leukemia. *N Engl J Med.* 2013;368(22):2059–2074. 10.1056/NEJMoa1301689 23634996 PMC3767041

[4] Papaemmanuil E Gerstung M Bullinger L: Genomic Classification and Prognosis in Acute Myeloid Leukemia. *N Engl J Med.* 2016;374(23):2209–2221. 10.1056/NEJMoa1516192 27276561 PMC4979995

[5] Barbie DA Tamayo P Boehm JS: Systematic RNA interference reveals that oncogenic *KRAS*-driven cancers require TBK1. *Nature.* 2009;462(7269):108–12. 10.1038/nature08460 19847166 PMC2783335

[6] Hänzelmann S Castelo R Guinney J: GSVA: gene set variation analysis for microarray and RNA-seq data. *BMC Bioinformatics.* 2013;14(1):7. 10.1186/1471-2105-14-7 23323831 PMC3618321

[7] Tomfohr J Lu J Kepler TB: Pathway level analysis of gene expression using singular value decomposition. *BMC Bioinformatics.* 2005;6(1):225. 10.1186/1471-2105-6-225 16156896 PMC1261155

[8] Lee E Chuang HY Kim JW: Inferring pathway activity toward precise disease classification. *PLoS Comput Biol.* 2008;4(11):e1000217. 10.1371/journal.pcbi.1000217 18989396 PMC2563693

[9] Foroutan M Bhuva DD Lyu R: Single sample scoring of molecular phenotypes. *BMC Bioinformatics.* 2018;19(1):404. 10.1186/s12859-018-2435-4 30400809 PMC6219008

[10] Handschuh L: Not Only Mutations Matter: Molecular Picture of Acute Myeloid Leukemia Emerging from Transcriptome Studies. *J Oncol.* 2019;2019:7239206. 10.1155/2019/7239206 31467542 PMC6699387

[11] Brunetti L Gundry MC Sorcini D: Mutant NPM1 Maintains the Leukemic State through HOX Expression. *Cancer Cell.* 2018;34(3):499–512.e9. 10.1016/j.ccell.2018.08.005 30205049 PMC6159911

[12] Verhaak RG Goudswaard CS van Putten W: Mutations in nucleophosmin *(NPM1)* in acute myeloid leukemia (AML): association with other gene abnormalities and previously established gene expression signatures and their favorable prognostic significance. *Blood.* 2005;106(12):3747–3754. 10.1182/blood-2005-05-2168 16109776

[13] Hess JL: *Mll*, *hox* genes, and leukemia: the plot thickens. *Blood.* 2004;103(8):2870–2871. 10.1182/blood-2004-01-0323

[14] Ross ME Mahfouz R Onciu M: Gene expression profiling of pediatric acute myelogenous leukemia. *Blood.* 2004;104(12):3679–3687. 10.1182/blood-2004-03-1154 15226186

[15] de Thé H Lavau C Marchio A: The PML-RAR alpha fusion mRNA generated by the t(15;17) translocation in acute promyelocytic leukemia encodes a functionally altered RAR. *Cell.* 1991;66(4):675–684. 10.1016/0092-8674(91)90113-D 1652369

[16] Ma X Liu Y Liu Y: Pan-cancer genome and transcriptome analyses of 1,699 paediatric leukaemias and solid tumours. *Nature.* 2018;555(7696):371–376. 10.1038/nature25795 29489755 PMC5854542

[17] Liberzon A Birger C Thorvaldsdóttir H: The Molecular Signatures Database (MSigDB) hallmark gene set collection. *Cell Syst.* 2015;1(6):417–425. 10.1016/j.cels.2015.12.004 26771021 PMC4707969

[18] Colaprico A Silva TC Olsen C: *TCGAbiolinks*: an R/Bioconductor package for integrative analysis of TCGA data. *Nucleic Acids Res.* 2016;44(8):e71. 10.1093/nar/gkv1507 26704973 PMC4856967

[19] Chen Y Lun AT Smyth GK: From reads to genes to pathways: differential expression analysis of RNA-Seq experiments using Rsubread and the edgeR quasi-likelihood pipeline [version 2; peer review: 5 approved]. *F1000Res.* 2016;5:1438. 10.12688/f1000research.8987.2 27508061 PMC4934518

[20] Law CW Alhamdoosh M Su S: RNA-seq analysis is easy as 1-2-3 with limma, Glimma and edgeR [version 3; peer review: 3 approved]. *F1000Res.* 2016;5: pii: ISCB Comm J-1408. 10.12688/f1000research.9005.3 27441086 PMC4937821

[21] Oshlack A Wakefield MJ: Transcript length bias in RNA-seq data confounds systems biology. *Biol Direct.* 2009;4(1):14. 10.1186/1745-6150-4-14 19371405 PMC2678084

[22] Zhao S Zhang B: A comprehensive evaluation of ensembl, RefSeq, and UCSC annotations in the context of RNA-seq read mapping and gene quantification. *BMC Genomics.* 2015;16(1):97. 10.1186/s12864-015-1308-8 25765860 PMC4339237

[23] Wu PY Phan JH Wang MD: Assessing the impact of human genome annotation choice on RNA-seq expression estimates. *BMC Bioinformatics.* 2013;14 Suppl 11:S8. 10.1186/1471-2105-14-S11-S8 24564364 PMC3816316

[24] Wang C Taciroglu A Maetschke SR: mCOPA: analysis of heterogeneous features in cancer expression data. *J Clin Bioinformas.* 2012;2(1):22. 10.1186/2043-9113-2-22 23216803 PMC3553066

[25] Foroutan M Cursons J Hediyeh-Zadeh S: A Transcriptional Program for Detecting TGFβ-Induced EMT in Cancer. *Mol Cancer Res.* 2017;15(5):619–631. 10.1158/1541-7786.MCR-16-0313 28119430

[26] Cursons J Pillman KA Scheer KG: Combinatorial Targeting by MicroRNAs Co-ordinates Post-transcriptional Control of EMT. *Cell Syst.* 2018;7(1):77–91.e7. 10.1016/j.cels.2018.05.019 30007539

