# Supplementary material for: Using singscore to predict mutation status in acute myeloid leukemia from transcriptomic signatures
Source: F1000Res. 2019 Oct 14;8:776. Originally published 2019 Jun 3. [Version 3] doi: 10.12688/f1000research.19236.3 (PMC6844140; doi:10.12688/f1000research.19236.3)
Supplement: Peer review history

# Peer review: Reviewer response for version 3

**DOI:** 10.5256/f1000research.22970.r55103

**Published:** 2019-11-06

**Authors:** Luiza Handschuh

**Affiliations:** 1 Institute of Bioorganic Chemistry, Polish Academy of Sciences (PAN), Poznań, Poland

All my comments were addressed by the authors. Some of the suggested improvements were introduced, other not but the authors explained they did not overlap with the objectives of the article.

 I only suggest to correct a few editorial errors:

- "Normlisation” instead of "Normalisation” occurs once in the chapter “Transformation to FPKM values and normalisation”;
- "those those” – repeated word in the sentence started from “Figure 2 shows that the set of down-regulated genes has more discriminant power…”
- there is no dot at the end of the last sentence of the Summary chapter. Moreover, I suggest not to use a term “wild-type NPM1c samples” formed as an equivalent of “NPM1c mutated samples”, e.g. in the description of the Figure 3 and 4, as “NPM1c” means “cytoplasmic NPM1” which is typical for samples with the mutated *NPM1* gene. The term “wild-type NPM1 samples” is more appropriate.

I confirm that I have read this submission and believe that I have an appropriate level of expertise to confirm that it is of an acceptable scientific standard.

# Peer review: Reviewer response for version 2

**DOI:** 10.5256/f1000research.22103.r52512

**Published:** 2019-08-15

**Authors:** Marina Lizio

**Affiliations:** 1 Division of Genomic Technologies, RIKEN Center for Life Science Technologies, Yokohama, Japan

I don't have many more comments.

 Figures could be improved, as noted in the first version of this peer review.

I confirm that I have read this submission and believe that I have an appropriate level of expertise to confirm that it is of an acceptable scientific standard.

# Peer review: Reviewer response for version 1

**DOI:** 10.5256/f1000research.21079.r51547

**Published:** 2019-08-05

**Authors:** Luiza Handschuh

**Affiliations:** 1 Institute of Bioorganic Chemistry, Polish Academy of Sciences (PAN), Poznań, Poland

**Introduction**

 The article presents a new package, which was developed from a previous one (‘singscore’) elaborated by the same authors. The package ‘singscore’ is a single-sample gene set scoring method which is valuable for analysis of transcriptomes of samples collected through the long time and not sequenced in the same run, experiment, platform or laboratory. Here, the authors apply the method for classification of TCGA acute myeloid (AML) samples using transcriptional ‘gene signatures’ identified by other authors (Verhaak and Ross) as typical for the NPM1c mutation, KMT2A (MLL) gene fusions, and PML-RARA gene fusions.

 AML is a heterogeneous and multi-clonal disease. The transcriptome picture of AML is very complex and can result from different mutations, genomic rearrangements, and aberrant regulation of gene expression at different levels (see my last review paper ^1^). Sometimes, the gene expression profiles can overlap between samples with different genetic lesions. The examples are HOX-gene-based discriminative signatures determined by Verhaak and Ross, not limited to AML with mutated NPM1, but specific also for AML cases with 11q23 abnormalities and KMT2A (MLL) gene rearrangements. The authors are aware of this fact and underline it in the article, too. On the other side, samples with the same mutation can present varying expression profiles because of additional features which also affect the transcriptome. Therefore, interpretation of the results must be done with caution. For me, the authors interpret the results a little bit too optimistically. The presented method is not sufficient to determine the mutation of interest, but can be used as a supplementary approach, a screening tool or (in future) a personalized medicine tool which could classify patients based on transcriptomic profiles, associated with specific treatment response.

**Testing package**

 I have used the package in R with TCGA data and confirm the code works, it is fast and generates the same results and plots as presented in the article. Moreover, I have tested the whole procedure on my own RNA-seq dataset (not published yet but used for supplementary expression analysis of NPM1 alternative transcripts, see the article ^2^). My dataset contains 28 AML samples, including 8 with NPM1 mutation (verified with three independent approaches). The data load was more tricky as I started from the csv table with counts and I had to convert the DESeqDataSet object into the RangedSummarizedExperiment object. The best discrimination was achieved with the Verhaak signature, but only with the down-regulated genes. All samples with NPM1 mutation were clearly separated from others, however, 2 additional samples, without NPM1 mutation, were grouped together with NPM1c. It is possible they have KMT2A (MLL) rearrangements, but I cannot verify it now. Analyzing the plots, up-regulated genes and all genes form the signature are not as efficient, separating only 2 or 3 NPM1c samples from the rest. In the article, the authors also admit that the set of down-regulated genes has more discriminant power, but they claim up-regulated genes also contribute to the discrimination.

 I have no samples with PML-RARA fusion in my dataset and the status of KMT2A (MLL) gene was unknown in my patients, so I could not compare the efficiency of signatures other than NPM1c. APL with PML-RARA is the most distinctive AML subtype which is easily separated from other AML samples based on transcriptome profile, so I would expect good results. This example shows the package works well for samples with very specific gene expression profile.

**Interpretation of the results**

 Considering the result interpretation, I have some doubts. The singscores are composed of two components, an enrichment score and a dispersion estimate of ranks. I am informed that “high expression of up-regulated genes and low expression of down-regulated genes would result in higher scores”. This is logical. I also know the maximal ranges ([−1, 1] for signatures involving up- and down-regulated genes). However, which value is really high? For example, is 0.2 enough (it looks like from the plots in the article and from my own data, too) or maybe I should expect much more, e.g. 0.7? Similarly, which value of dispersion should I expect? I suppose, the lowest, but which is low enough or which range is optimal? What is difficult to understand is that “despite the range of scores increasing, the discriminant power drops moderately” (for up-regulated genes from Verhaak signature, when compared to down-regulated genes). I see the same paradox in my data – the scores are higher for up-reg. genes, which are much less efficient in discrimination between NPM1c+ and NPM1c- samples. It looks like scores only do not reflect the trends observed from score vs dispersion plots.

**Possible improvements**

 Although the package is generally useful and well described, the following improvements will make it more friendly for less advanced R users, e.g.

- Instruction how to prepare and load the data other than those deposited in the GDC database.

- Annotation of samples on the plots with unique sample IDs (when a sample is misclassified, a user does not know which one it is; it will also be helpful to localize a sample on various plots, e.g. generated with a signature of up- and down-regulated genes) – it is written in the article that the ‘singscore’ package supports different types of annotations (“Annotations of interest can be overlayed on each plot”) but I found only color code annotation whereas I would like to have color code for mutation type and additional text labels with sample IDs on the same plot.
- A command which lists the samples typed as strong candidates for having particular mutation, ordered according to the calculated metrics.
- Generating a threshold line between samples with and without mutation.

In future, it would be also good to include other signatures, e.g. the signature of 369 genes identified by Alcalay *et al*. in 2005 ^3^, discriminating AML patients with NPMc+ from patients with NPMc-, even in cases with rare chromosomal abnormalities.

 What I would expect the most from a package designed to identify mutations in transcriptomes, is a direct identification of mutations in RNAseq data. The results of mutation typing based on gene expression profile will be strongly supported when a particular mutation will be covered by RNAseq reads. From my own experience, I know it is possible (for NPM1 mutation detection, see ^4^). For genes with high and middle expression level, the coverage can be even higher than that obtained from genome- or exome-level data. And in the case when DNA data are unavailable, it would be really fantastic. Because it demands completely different data processing, it may be considered for future versions of the package.

I confirm that I have read this submission and believe that I have an appropriate level of expertise to confirm that it is of an acceptable scientific standard, however I have significant reservations, as outlined above.

## References

[1] : Not Only Mutations Matter: Molecular Picture of Acute Myeloid Leukemia Emerging from Transcriptome Studies. *Journal of Oncology*.2019;2019: 10.1155/2019/7239206 1-36 10.1155/2019/7239206 PMC669938731467542

[2] : NPM1 alternative transcripts are upregulated in acute myeloid and lymphoblastic leukemia and their expression level affects patient outcome. *Journal of Translational Medicine*.2018;16(1) : 10.1186/s12967-018-1608-2 10.1186/s12967-018-1608-2 30126426PMC6102803

[3] : Acute myeloid leukemia bearing cytoplasmic nucleophosmin (NPMc+ AML) shows a distinct gene expression profile characterized by up-regulation of genes involved in stem-cell maintenance. *Blood*.2005;106(3) : 10.1182/blood-2005-02-0560 899-902 10.1182/blood-2005-02-0560 15831697

[4] : Simultaneous detection of mutations and copy number variation of NPM1 in the acute myeloid leukemia using multiplex ligation-dependent probe amplification. *Mutat Res*.2016;786: 10.1016/j.mrfmmm.2016.02.001 14-26 10.1016/j.mrfmmm.2016.02.001 26894557

# Response

**Published:** 2019-10-14

**Authors:** Melissa Davis

**Affiliations:** The Walter and Eliza Hall Institute, Australia

*We thank the reviewer for their time and effort directed towards reviewing our manuscript and for the helpful feedback they have provided. Where appropriate we have modified our manuscript to address the reviewer’s comments.*

*Below we include a point by point response to the reviewer’s comments and where appropriate we list corresponding changes to the manuscript.*

**Introduction**

**LH**: The article presents a new package, which was developed from a previous one (‘singscore’) elaborated by the same authors. The package ‘singscore’ is a single-sample gene set scoring method which is valuable for analysis of transcriptomes of samples collected through the long time and not sequenced in the same run, experiment, platform or laboratory. Here, the authors apply the method for classification of TCGA acute myeloid (AML) samples using transcriptional ‘gene signatures’ identified by other authors (Verhaak and Ross) as typical for the NPM1c mutation, KMT2A (MLL) gene fusions, and PML-RARA gene fusions.

***DDB**: We note that the purpose of the initial manuscript may have been unclear, in part because it is listed under the F1000 Software Tool Article section. The purpose of this article is to present a workflow demonstrating the use of singscore, and it is a R/Bioconductor workflow illustrating the use of singscore, and so is not intended as a new package or tool.*

*https://www.bioconductor.org/packages/release/workflows/*

*https://www.bioconductor.org/packages/release/workflows/html/SingscoreAMLMutations.html*

*Some of the review comments below assume that this manuscript presents a specific software package for detecting NPM1 mutations, so we have clarified the manuscript as outlined below in order to make the purpose and intention more clear (note that the title of the manuscript has changed from ‘predicting mutations’ to ‘predicting mutation status’ based upon feedback from reviewer 1).*

*This work arose from an observation in another project that the Verhaak signature scored with singscore appeared to correlate strongly with mutation status, and we thought this would be an interesting example that might also help researchers to investigate the links between genetic lesions and corresponding transcriptional changes, an area in which the reviewer clearly has expertise.*

**LH**: AML is a heterogeneous and multi-clonal disease. The transcriptome picture of AML is very complex and can result from different mutations, genomic rearrangements, and aberrant regulation of gene expression at different levels (see my last review paper ^1^). Sometimes, the gene expression profiles can overlap between samples with different genetic lesions. The examples are HOX-gene-based discriminative signatures determined by Verhaak and Ross, not limited to AML with mutated NPM1, but specific also for AML cases with 11q23 abnormalities and KMT2A (MLL) gene rearrangements. The authors are aware of this fact and underline it in the article, too. On the other side, samples with the same mutation can present varying expression profiles because of additional features which also affect the transcriptome.

**LH**: Therefore, interpretation of the results must be done with caution. For me, the authors interpret the results a little bit too optimistically. The presented method is not sufficient to determine the mutation of interest, but can be used as a supplementary approach, a screening tool or (in future) a personalized medicine tool which could classify patients based on transcriptomic profiles, associated with specific treatment response.

***DDB*** *: We agree with the reviewer that gene set scoring of transcriptomic data should not be the only method used to identify patient samples carrying genetic lesions. The Bioconductor workflow we present is intended to provide an example of applying singscore to study mutation/fusion based gene sets as we believe that singscore provides a relatively flexible and intuitive approach for investigating different gene sets across large data sets. We feel that a particularly useful feature is the ability to combine different signatures/gene sets (including phenotype/cell-cycle signatures etc) to explore how these transcriptional changes are associated over different samples, demonstrated in Figures 5-7.*

*We have modified the “Description of biological relevance” section to address the reviewer’s comments around the complexity of AML genomic lesions and corresponding transcriptomic changes. The review highlighted is particularly relevant for this work and accordingly we mention the complexity of AML and direct the reader towards this resource by extending the first paragraph (new text underlined):*

*.. A more recent study which has focused primarily on genomic data has further refined the clinically significant AML subtypes [Papaemmanuil (2016), NEJM], highlighting a number of co-occurring as well as mutually exclusive mutations.* *As the identification of putative driver fusions/mutations continues, work has also been directed towards how these lesions interact with each other and other features (e.g. cellular proliferation, changes due to phenotypic plasticity, or variation in post-transcriptional regulators such as microRNAs) to drive transcriptional changes as discussed in a recent review [Hanschuch (2019), J. Oncol.]* *.*

*We have also added a paragraph at the end of this section which discusses some of the limitations for our approach and provides more context in which it could be applied:*

*While we demonstrate that singscore is capable of inferring mutation status from the transcriptional profile of AML samples, we note that it is best used to supplement alternative data which can provide a more definitive resolution of these lesions. Processing of raw RNA‑seq data will directly identify the presence of gene‑fusion products or mutations within protein-coding regions, although for many large data sets the quantified transcript abundance data are much easier to obtain without access agreements. The method can also be applied to legacy microarray data sets where genome and RNA sequencing data are unavailable. As such singscore provides a useful approach to supplement established methods for the study of genetic lesions in cancer. By exploring associations between different genomic and phenotypically relevant signatures, it may also help to better characterise true driver mutations which exert consistent effects on the transcriptome of AML samples and other cancers.*

**Testing package**

**LH**: I have used the package in R with TCGA data and confirm the code works, it is fast and generates the same results and plots as presented in the article. Moreover, I have tested the whole procedure on my own RNA-seq dataset (not published yet but used for supplementary expression analysis of NPM1 alternative transcripts, see the article ^2^). My dataset contains 28 AML samples, including 8 with NPM1 mutation (verified with three independent approaches). The data load was more tricky as I started from the csv table with counts and I had to convert the DESeqDataSet object into the RangedSummarizedExperiment object. The best discrimination was achieved with the Verhaak signature, but only with the down-regulated genes. All samples with NPM1 mutation were clearly separated from others, however, 2 additional samples, without NPM1 mutation, were grouped together with NPM1c. It is possible they have KMT2A (MLL) rearrangements, but I cannot verify it now. Analyzing the plots, up-regulated genes and all genes form the signature are not as efficient, separating only 2 or 3 NPM1c samples from the rest. In the article, the authors also admit that the set of down-regulated genes has more discriminant power, but they claim up-regulated genes also contribute to the discrimination.

**LH**: I have no samples with PML-RARA fusion in my dataset and the status of KMT2A (MLL) gene was unknown in my patients, so I could not compare the efficiency of signatures other than NPM1c. APL with PML-RARA is the most distinctive AML subtype which is easily separated from other AML samples based on transcriptome profile, so I would expect good results. This example shows the package works well for samples with very specific gene expression profile.

***DDB*** *: We thank the reviewer for the exceptional effort and time invested to test our workflow on independent data - we hope results from this analysis have been informative for identifying other features within their data. Our workflow includes guidance for users who wish to import data from other sources such as those used by the reviewer.*

**Interpretation of the results**

**LH**: Considering the result interpretation, I have some doubts. The singscores are composed of two components, an enrichment score and a dispersion estimate of ranks. I am informed that “high expression of up-regulated genes and low expression of down-regulated genes would result in higher scores”. This is logical. I also know the maximal ranges ([−1, 1] for signatures involving up- and down-regulated genes). However, which value is really high? For example, is 0.2 enough (it looks like from the plots in the article and from my own data, too) or maybe I should expect much more, e.g. 0.7? Similarly, which value of dispersion should I expect? I suppose, the lowest, but which is low enough or which range is optimal? What is difficult to understand is that “despite the range of scores increasing, the discriminant power drops moderately” (for up-regulated genes from Verhaak signature, when compared to down-regulated genes). I see the same paradox in my data – the scores are higher for up-reg. genes, which are much less efficient in discrimination between NPM1c+ and NPM1c- samples. It looks like scores only do not reflect the trends observed from score vs dispersion plots.

***DDB**: Interpretation of singscores is intentionally left to be problem specific as it generally requires some domain-specific knowledge of the biological system and corresponding signature genes – ideally the computational biologist or bioinformaticians working on each project can provide some guidance.*

*The basic interpretation of the score is the normalised mean rank of genes within the signature relative to all other genes in the sample. At the extrema this interpretation is relatively simple - near 1, a higher value would indicate that genes in the signature are expressed at higher levels relative to other genes. For scores towards zero, however, the interpretation can be much more difficult – a score of zero (on the range [-1,1]) could indicate the signature genes are tightly clustered around the sample-wide mean abundance, or it could indicate a highly-dispersed almost uniform distribution across the entire abundance range (with a mean near the mean of all genes). By exploring the singscores together with dispersion estimates this information is summarised, assisting in estimation of effect size variability.*

*Interpretation of scores depends on the context of the experiment and the typical behaviour of the gene set. A “high” score is best determined relative to other samples. This is achieved either by comparing scores from other samples in large datasets such as TCGA, or better, across a set of samples from a given experiment with known conditions. Other methods normalise the data before computing scores, and we note that a recent paper has applied z-score normalisation to results from singscore for comparison to ssGSEA [Cui et al (2019) Oncogene, DOI: 10.1038/s41388-019-1026-9].*

*All singscores for samples remain the same and do not have to be recomputed upon addition of new samples, and interpretation will improve as more samples are added to the study. For example, Gaussian mixture modelling could be used to separate the NPM1c scores based on our expectation that there are two groups. This could be switched with other unsupervised classification algorithms such as hierarchical clustering or k-means clustering. We have added an example analysis to the manuscript to demonstrate how scores can be interpreted in an unsupervised setting, under the section “Transcriptional signatures to predict mutation status/Unsupervised classification of mutations”.*

*There may be some cases where sample annotation is not available. In such scenarios, we are unable to build regression models to interpret scores. A higher singscore would provide stronger evidence for the signature but the magnitude is difficult to interpret without a reference. One approach to deal with this situation is to compare scores to those from other datasets where the mutations status is known. An alternative approach would be to compare scores within the dataset using unsupervised learning methods.*

*Here we demonstrate the use of three clustering methods (Gaussian mixture decomposition, k-means clustering, and hierarchical clustering) to stratify samples, and as we have done previously [wang et al (2012) Journal of clinical bioinformatics] use the adjusted Rand index (ARI) to compare classifications. As expected, supervised (GLM) classification results in the best prediction. This is followed by clustering based on the score using Gaussian mixture decomposition. Any other classification algorithm along with prior knowledge could be used to decompose scores into groups. The important characteristic of singscores is that they maintain the discriminant power of gene signatures therefore can be coupled with supervised, semi-supervised or unsupervised algorithms to perform stratification.*

*```*

*library(mclust)*

*#Gaussian mixture model*

*m1 = Mclust(scoredf$Score, G = 2, verbose = FALSE)*

*#k-means clustering*

*m2 = kmeans(scoredf[, 5:6], centers = 2, nstart = 100)*

*#hierarchical clustering*

*m3 = hclust(dist(scoredf[, 5:6]))*

*mutation_inference = cbind(*

*'GLM' = prediction,*

*'mclust' = m1$classification,*

*'k-means' = m2$cluster,*

*'hclust' = cutree(m3, k = 2)*

*)*

*apply(mutation_inference, 2, adjustedRandIndex, scoredf$NPM1c.Mut)*

*```*

**Possible improvements**

**LH**: Although the package is generally useful and well described, the following improvements will make it more friendly for less advanced R users, e.g.

**LH**: Instruction how to prepare and load the data other than those deposited in the GDC database.

***DDB**: We have noted in text that the rank matrix can be computed using either a SummarizedExperiment object, DGEList object, ExpressionSet object, numeric matrix or a numeric data frame. As such, a numeric matrix with sample names as column names and genes as row names would suffice. Scoring should be performed on length bias corrected measurements such as RPKM/FPKM or TPM and not CPM or raw counts.*

*Transcriptional signatures to predict mutation status/Score TCGA AML samples using the Verhaak signature - extract from text: “The `rankGenes` function will compute ranks from expression data in the form of either a numeric matrix, numeric data frame, ExpressionSet object, DGEList object or a SummarizedExperiment object”*

**LH**: Annotation of samples on the plots with unique sample IDs (when a sample is misclassified, a user does not know which one it is; it will also be helpful to localize a sample on various plots, e.g. generated with a signature of up- and down-regulated genes) – it is written in the article that the ‘singscore’ package supports different types of annotations (“Annotations of interest can be overlayed on each plot”) but I found only color code annotation whereas I would like to have color code for mutation type and additional text labels with sample IDs on the same plot.

***DDB**: Sample labels can be added to landscape plots but were not supported in other visualisations. We have added functionality to the latest version of the singscore package (v1.5.1) to allow labelling of samples in the score vs. dispersion plots. We have modified the text to clarify, and modified Figure 6 to label samples where classification uncertainty (NMP1c vs WT) is high to demonstrate this feature. The changes below have been made to the section: “Transcriptional signatures to predict mutation status/Diagnostics of the Verhaak signature”.*

*… Sample annotations of interest (e.g. clinical annotations) can be colour coded on each plot. …*

***Figure 6:***

*```*

*select_aml = !mutated_gene %in% 'Other'*

*#label samples with an mclust NPM1c classification uncertainty of > 0.3*

*label_samples = substr(rownames(verhaak_scores), 6, 12) #sample ID from barcodes*

*label_samples[m1$uncertainty < 0.3] = NA*

*#project mutations onto the landscape*

*p1 = projectScoreLandscape(*

*p_mll_npm1c,*

*verhaak_scores,*

*rossmll_scores,*

*subSamples = select_aml,*

*annot = mutated_gene[select_aml],*

*sampleLabels = label_samples[select_aml]*

*)*

*p1 + theme(legend.box = 'vertical')*

*```*

**LH**: A command which lists the samples typed as strong candidates for having particular mutation, ordered according to the calculated metrics.

***DDB**: As discussed in an earlier comment, we recommend such analyses to be problem specific. Generally, a higher score would indicate a stronger effect of genes in the signature relative to WT samples therefore samples with higher scores would be stronger candidates for mutations. Alternatively, the partitioning created using Gaussian mixture modelling could be used as a guide for separation and samples with a score much higher than the threshold would be the strongest candidates for the mutation.*

**LH**: Generating a threshold line between samples with and without mutation.

***DDB**: See above discussion/recommendation.*

**LH**: In future, it would be also good to include other signatures, e.g. the signature of 369 genes identified by Alcalay *et al*. in 2005 ^3^, discriminating AML patients with NPMc+ from patients with NPMc-, even in cases with rare chromosomal abnormalities.

**LH**: What I would expect the most from a package designed to identify mutations in transcriptomes, is a direct identification of mutations in RNAseq data. The results of mutation typing based on gene expression profile will be strongly supported when a particular mutation will be covered by RNAseq reads. From my own experience, I know it is possible (for NPM1 mutation detection, see ^4^). For genes with high and middle expression level, the coverage can be even higher than that obtained from genome- or exome-level data. And in the case when DNA data are unavailable, it would be really fantastic. Because it demands completely different data processing, it may be considered for future versions of the package.

***DDB**: As we have outlined at the start of this review there may have been some misunderstanding around the purpose of this Workflow package/paper. We agree with the reviewer that direct detection of mutations/fusions from RNA-seq data is the best approach, and we now note this in the ‘Description of biological relevance section’ as stated above. The other gene signatures mentioned above could be incorporated in a workflow as singscore supports analysis and comparison of multiple gene sets.*

# Peer review: Reviewer response for version 1

**DOI:** 10.5256/f1000research.21079.r49410

**Published:** 2019-06-04

**Authors:** Marina Lizio

**Affiliations:** 1 Division of Genomic Technologies, RIKEN Center for Life Science Technologies, Yokohama, Japan

The authors Bhuva et al. presented the application of a previously published gene set scoring tool, singscore, to discriminate AML subsets based on gene expression signatures, using signatures derived from known specific gene mutations or fusions. The authors show that singscore can effectively separate AML samples based on such gene signatures.

 Given the ability of the software to discriminate samples within heterogenous diseases, such as leukemias, its application to other cancers and diseases with similar levels of heterogeneity can be beneficial to improve patients' clinical outcomes.

 The rationale behind the application of the software and its step-wise descriptions are well explained. So is the interpretation of results.

 I appreciate the authors' efforts to explain all the steps and suggest alternatives, giving the software description the feel of a tutorial.

 I have some small comments on some points I'd like to see addressed:

1. Title. As far as I have understood, singscore doesn't actually predict mutations. Rather, it uses gene signatures associated to certain mutations in order to identify sub-groups within a population. As it is, the title is misleading.
2. Similar rationale about the title of section "Predicting mutations using the Verhaak signature", page 16.
3. In the section "Filter out genes with low counts", page 7, the authors describe explicitly how to handle duplicated entries caused by the GDCprepare function. Here I would reorganise and shorten the paragraph by first mentioning the issue introduced by the function, then saying edgeR is applied to the data filtered of duplicate entries.
4. Figure 3. The legend below each graph is identical. I suggest to only use one legend for all three plots.
5. The authors comment on several instances about the similarities between scores for signatures of two subgroups of AML samples, NPM1c and KMT2A-/MLL-fusion, and that's due to the presence of Hox genes. I suggest to have the lists of the two gene signatures directly in the article for immediate lookup. This is not essential, as the authors already refer to the articles they take the data from, but I think it will help readers.

Minor comments: 

1. Typo in the caption of figure 1 (approcimately instead of approximately).
2. Typo in figure 2 caption. I suppose "scores are plot against..." should read "scores are plotted against...".

I confirm that I have read this submission and believe that I have an appropriate level of expertise to confirm that it is of an acceptable scientific standard.

# Response

**Published:** 2019-08-16

**Authors:** Melissa Davis

**Affiliations:** The Walter and Eliza Hall Institute, Australia

We would like to thank Dr Marina Lizio for her very useful review, and we have addressed her comments as outlined below.

**ML: I have some small comments on some points I'd like to see addressed:**

 1. Title. As far as I have understood, singscore doesn't actually predict mutations. Rather, it uses gene signatures associated to certain mutations in order to identify sub-groups within a population. As it is, the title is misleading.

*DDB: This is an excellent point. We have now changed the title to “Using singscore to predict mutation **status** in AML from transcriptomic signatures” in order to clarify that we are simply predicting the mutation status of samples, not actual mutations.*

 2. Similar rationale about the title of section "Predicting mutations using the Verhaak signature", page 16.

*DDB: A similar change is made here, the title of this section is now: “Predicting **NPM1c mutation status** using the Verhaak signature”.*

 3. In the section "Filter out genes with low counts", page 7, the authors describe explicitly how to handle duplicated entries caused by the GDCprepare function. Here I would reorganise and shorten the paragraph by first mentioning the issue introduced by the function, then saying edgeR is applied to the data filtered of duplicate entries.

*DDB: The issue of duplicated features with the TCGAbiolinks package has been fixed in a recent update (TCGAbiolinks v2.12.2). Consequently, we will be removing the text describing the identification and removal of duplicated features. The text removed is:*

*------------------------------*

*Creation of a DGEList object is initially unsuccessful due to duplicated row names in the data matrix (107 features). The raw count files for individual samples do not have any duplicated features indicating this is introduced by the `GDCprepare` function. This is further supported by the fact that the counts for a single feature are the same for duplicated entries. An example of such a feature is shown below and we have verified duplicated entries of all other features to be the same.*

*```{r}*

*dup_features = rownames(aml_se)[duplicated(aml_se)]*

*```*

*```{r dup_entries}*

*library(SummarizedExperiment)*

*#example check on one feature ID*

*dup_feature = 'ENSG00000011454'*

*dup_data = assay(aml_se[rownames(aml_se) %in% dup_feature, ])*

*identical(dup_data[1, ], dup_data[2, ])*

*```*

*As such, it is safe to discard duplicated entries.*

*------------------------------*

*We also remove the code that discards duplicate features*

*------------------------------*

*aml_se = aml_se[!duplicated(rownames(aml_se)), ]*

*------------------------------*

 4. Figure 3. The legend below each graph is identical. I suggest to only use one legend for all three plots.

*DDB: We appreciate this point, however each plot in this figure is generated by a function in the package which includes the legend. For a normal use case, users would be generating a single plot to diagnose a single sample, which is why we include a legend for the plot. In this workflow, we simply combine the three plots into a single figure. Removing legends from the default plots would overcomplicate the code of this diagnostic procedure in the workflow which is why we decided to leave the legends as they are. If users were interested in manipulating figures in their application, they could add extra options to the ggplot2 object (as we show with adding a new title and modifying the legend to be a single column).*

 5. The authors comment on several instances about the similarities between scores for signatures of two subgroups of AML samples, NPM1c and KMT2A-/MLL-fusion, and that's due to the presence of Hox genes. I suggest to have the lists of the two gene signatures directly in the article for immediate lookup. This is not essential, as the authors already refer to the articles they take the data from, but I think it will help readers.

*DDB: The signatures we downloaded from MSigDB have Entrez IDs for genes. The NPM1c and MLL signatures contain 491 unique genes. The reason we did not have the lists in the article was that we would be presenting 491 numbers that would have been difficult to interpret without mapping. Additionally, our intention is for readers to execute the workflow while reading this article. In this scenario, the geneIds() function could be used to list the gene identifiers forming the signatures once they have been downloaded and parsed into R. Identifiers could be mapped to symbols using the mapIds() function in the AnnotationDbi package and an annotation such those provided by the org.Hs.eg.db R/Bioconductor package. We hope that the ease with which these data can be accessed in the application will make the lists accessible for readers.*

**ML: Minor comments:**

 1. Typo in the caption of figure 1 (approcimately instead of approximately).

*DDB: Thanks for helping us identify this, we have fixed it now.*

 2. Typo in figure 2 caption. I suppose "scores are plot against..." should read "scores are plotted against...".

*DDB: This has also been changed.*

 Many thanks for the useful and constructive review.

 Best wishes,

 Mr Dharmesh Bhuva

 Bioinformatics Division

 The Walter and Eliza Hall Institute

 Dr Melissa Davis

 Bioinformatics Division

 The Walter and Eliza Hall Institute
